# LesionSCynth: A simple parametric lesion synthesis method to improve spinal cord lesion segmentation in low-data scenarios

**DOI:** 10.1162/IMAG.a.1029

**Published:** 2025-11-26

**Authors:** Ricky Walsh, Prabhjot Kaur, Davood Karimi, Anne Kerbrat, Simon K. Warfield, Francesca Galassi, Benoit Combès

**Affiliations:** Univ Rennes, Inria, CNRS, Inserm, IRISA UMR 6074, Empenn, Rennes, France; Computational Radiology Laboratory, Boston Children’s Hospital and Harvard Medical School, Boston, MA, United States; Department of Neurology, Rennes University Hospital, Rennes, France

**Keywords:** synthetic data, multiple sclerosis, spinal cord, lesion segmentation, few annotations

## Abstract

Detecting multiple sclerosis lesions in spinal cord MRI is a critical but complex task for radiologists and neurologists. While deep learning models have shown promise for this task, training these models requires large manually annotated datasets, which are time consuming and expensive to create. To reduce the annotation burden, this study proposes LesionSCynth, a parametric framework for synthesising hyperintense lesions in spinal cord MRI. Based on analysis of the intensity distribution of real lesions in sagittal T2-weighted acquisitions, LesionSCynth generates realistic synthetic lesions that can augment small annotated datasets. Segmentation models trained on a combination of these synthetic lesions and 17 real acquisitions achieved notably better performance than those trained on the real lesions alone (0.52 vs. 0.46 FROC, respectively). Notably, models trained with LesionSCynth lesions achieved similar detection performance to a model trained on eight times more real lesions (0.55 FROC for both). Moreover, LesionSCynth outperformed two leading lesion synthesis methods, LesionMix (0.43) and CarveMix (0.41), in low-data regimes. These findings position LesionSCynth as a practical and effective solution for reducing the annotation burden while improving multiple sclerosis (MS) lesion detection and segmentation in spinal cord MRI.

## 1 Introduction

### 1.1 Motivation

Multiple sclerosis (MS) is a chronic demyelinating disease of the central nervous system, and is a leading cause of disability in young adults (Jakimovski et al., 2024). A key diagnostic criterion for MS is the presence of white matter lesions in the brain and spinal cord, which appear as hyperintense in T2-weighted (T2-w) magnetic resonance (MR) scans (Thompson et al., 2018). However, accurately identifying these lesions is a complex and mentally demanding task for radiologists and neurologists, and is prone to significant inter-rater variability (Lodé et al., 2025; Walsh et al., 2023).

Automated tools, particularly those based on deep learning, have demonstrated impressive performance for this task, and have been shown to improve the sensitivity of experts for both brain and spinal cord lesions (Combès et al., 2021; Lodé et al., 2025). However, automated lesion segmentation in spinal cord MRI has remained relatively less studied compared with the brain. The narrow, elongated, and mobile anatomy of the spinal cord introduces additional challenges, such as a higher susceptibility to motion and respiratory artefacts, as well as partial volume effects with cerebrospinal fluid (CSF) (Kearney et al., 2015). Moreover, heterogeneity of acquisition protocols for spinal cord MRI, where many different MR sequences can be used for acquisitions (Wattjes et al., 2021), complicates the development of generalisable deep learning models. Training such models requires large, annotated datasets covering the whole range of acquisition protocols, which are often difficult to obtain.

One way to reduce the annotation burden is to synthesise entire images or to insert synthetic lesions into existing ones (Brugnara et al., 2024; Ferreira et al., 2024; Terrassin et al., 2024). Although generative adversarial networks (GANs) and diffusion models create realistic synthetic images, they require large annotated datasets for training, so are not applicable in low-data scenarios. Several non-model-based techniques have been proposed for lesion synthesis, such as CarveMix (X. Zhang et al., 2023) and LesionMix (Basaran et al., 2024). However, these methods typically rely on using existing lesions from the training dataset, and so may overfit to the specific intensity characteristics, especially when only a small number of real lesions are available.

This study proposes *LesionSCynth*, a parametric method for lesion synthesis in spinal cord MRI, designed to improve the performance of deep learning segmentation models in low-data scenarios, thereby reducing the annotation demand. Based on an analysis of spinal cord lesion characteristics, our approach generates synthetic lesions using real lesion shapes and a modelled hyperintensity profile. During training, we insert synthetic lesions into real MR volumes and use these alongside a small set of real annotated lesions, to aid the model to generalise to a wider distribution of lesion intensity characteristics. The study demonstrates that a simple parametric method incorporating basic priors can lead to impressive improvements in low-data scenarios. Moreover, although the focus here is on spinal cord lesions in sagittal T2-weighted acquisitions, the method could be easily adapted to generate other types of hyperintense or hypointense lesions and to be used in other imaging modalities.

The contributions of the study are threefold: (1) a parametric method of lesion synthesis, which allows a greater variability of intensity characteristics in low-data scenarios and outperforms state-of-the-art lesion synthesis methods—to the best of our knowledge, this is the first study of lesion synthesis for spinal cord lesion segmentation; (2) comprehensive investigations and ablation studies to understand the impact of various design choices in the proposed method; and (3) a thorough evaluation of the effect of the proportion of images with lesions versus without lesions, and the ratio of real lesions versus synthetic lesions, across several different training set sizes.

The paper is organised as follows: the next subsection discusses related literature in MS lesion segmentation in spinal cord MRI and in lesion synthesis. Section 2 describes the dataset and preprocessing used in this study. Section 3 details the lesion synthesis method and the empirical observations on which it is based. Section 4 describes the experiments undertaken, while Section 5 presents the results of these experiments. Finally, the results and outlook are discussed in Section 6.

### 1.2 Related work

#### 1.2.1 MS spinal lesion segmentation

While MS lesion segmentation in brain MRI has been widely studied over the years, with many challenges and open datasets (Carass et al., 2017; Commowick et al., 2016, 2021; Lesjak et al., 2018), spinal cord MRI has remained less studied. A major contribution in this area came from Gros et al. (2019), where the authors trained deep learning models to segment mainly cervical spinal cord MS lesions in axial and sagittal T2-w volumes. These models were then released in the open-source Spinal Cord Toolbox (SCT). However, some limitations were identified over time, for example, poor performance when applied to the thoracic and lumbar spinal cord (Karthik et al., 2025). Recent updates have improved upon this previous version, including a contrast-agnostic model and a model for axial T2-w scans (Karthik et al., 2025). In all cases, training the models required hundreds or even thousands of examples with segmentation masks manually or semi-automatically generated by expert human raters. Such an effort, however, is not always feasible due to time and budget constraints.

Recent studies have begun to explore frameworks that account for the wide range of possible acquisition sequences and resulting variability in image contrast (Benveniste et al., 2025; Walsh et al., 2024). While the approach taken by Walsh et al. (2024) was to use the manual segmentation masks from the T2-w volumes for the corresponding sequences, this is suboptimal as there can be misalignments between the acquisitions, differences in coverage, and the visible extent of the lesion can vary across acquisitions because of limited resolution, partial volume effects, and artefacts. Therefore, to avoid introducing label noise, a set of high-quality manual annotations would be required for each distinct image contrast, which would further increase the annotation burden.

Given the increasing requirements for time-consuming and expensive manual annotations, the current study proposes a parametric method for lesion synthesis which, given a small set of real annotated lesions, can improve the performance of a deep learning segmentation model.

#### 1.2.2 Image synthesis

Deep learning methods have represented the state of the art in image synthesis since the advent of generative adversarial networks (GAN) (Goodfellow et al., 2014) and more recently, diffusion-based models have significantly improved synthetic image generation (Ho et al., 2020). These methods have been applied to numerous medical imaging tasks as a data augmentation technique, for example, for MS brain lesion segmentation (Brugnara et al., 2024), brain tumours (Ferreira et al., 2024), and kidney and liver tumours (Chen et al., 2024). However, training these models requires a large amount of annotated images and so they are not applicable for low-data scenarios.

Several non-model-based techniques have been inspired by the MixUp data augmentation technique (H. Zhang et al., 2018). Here, a new data sample is created by taking a linear combination of two existing samples. Although this may be effective for natural image classification, if we combine two medical images in this way, then we lose fine-grained information about local hyperintense regions which would render MixUp suboptimal for MS lesion segmentation. CutMix, based on a similar idea of combining two images from the training set, later achieved superior performance on ImageNet classification over MixUp (Yun et al., 2019). CutMix involves cutting out a random patch of one image and inserting a patch from another image into this gap, thus maintaining local continuity of information in the image, although creating clear artefacts at the patch boundary.

Several methods for lesion synthesis in medical images are largely inspired by this idea of cutting an area from one image and inserting it into another image in the training set (Basaran et al., 2024; X. Zhang et al., 2023; Zhu et al., 2022). CarveMix (X. Zhang et al., 2023) was developed to improve automated segmentation of brain lesions and tumours in MRI. CarveMix operates on whole images, by extracting all lesions in a given image and pasting them into another training image, maintaining the original lesion positions. The area copied from one image to another can be smaller or larger than the lesion mask, and this area is determined by the distance to the lesion boundary, with a target distance randomly sampled for each case.

LesionMix (Basaran et al., 2024) was developed in the context of automated segmentation of white matter hyperintensity in FLAIR MRI and of liver lesions in CT scans. The method includes two branches for data augmentation, one for inserting new lesions in an image, and another for removing existing lesions through inpainting. Given an existing image in the training set to be augmented, the total lesion load is first calculated and compared with a target load which is sampled randomly from some distribution (e.g., the true distribution of lesion load in the dataset, or a uniform distribution). If the current load is lower than the target load, then lesions from other images are iteratively inserted into the current image until the target load is reached. The inserted lesion positions are based on a prior spatial probability map. If the current load is higher than the target load, lesions in the current image are iteratively inpainted until below the target load.

A major drawback of CarveMix, LesionMix, and similar methods is that when only a small number of manual segmentations of real lesions are available, models may overfit to their intensity characteristics. Moreover, if the intensity profiles of the source and target images differ significantly, these methods can produce unrealistic lesions, or can even create areas of hypointensity where there should be a hyperintense synthetic lesion, which could inject significant noise into the learning process. The z-score normalisation applied by these methods to harmonise intensity distributions between images is not sufficient to eliminate these inverted lesions.

To address these limitations, we propose a method of lesion synthesis which generates lesions with diverse and controlled intensity profiles, even when the number of real annotated lesions is small. The intensity characteristics of the synthetic lesions can be controlled through the parameters of the method, thereby ensuring that inserted lesions are always hyperintense (or hypointense, if used for T1-w contrasts).

## 2 Data

### 2.1 Overview

The dataset used for the experiments in this study included 324 sagittal T2-w acquisitions corresponding to 215 unique patients, with 202 volumes of the upper spinal cord and 122 volumes of the lower spinal cord. The upper acquisitions covered the whole cervical spinal cord and several thoracic vertebrae (ending at T2-T9, depending on the acquisition). The lower acquisitions usually overlapped with the upper acquisitions by several vertebrae, and the coverage typically descended below the lumbar vertebrae.

Although this dataset does not represent a “low data” setting, it allowed us to examine the impact of lesion synthesis at different dataset sizes. Not all researchers have easy access to a dataset of this size, however, so our method could be directly applicable in those cases. Moreover, even if a dataset of this size is available, lesion synthesis may still be useful when attempting to improve generalisation of a segmentation model to other scanners, or even to different MRI sequences.

Approximately half of the data came from the French MS Registry (OFSEP) (Vukusic et al., 2020) and this was supplemented by acquisitions from three clinical studies: EMISEP (NCT02117375), MS-TRACTS (NCT04220814), and MAPMS (NCT04918225). Written informed consent was obtained from all participants for being included in the study. The study was compliant with French data confidentiality regulations.

The volumes were acquired with scanners from 3 brands, Siemens (84%), GE (10%), and Philips (6%), and 16 scanner models were represented in the dataset. The median in-plane dimension was 512 and the median number of slices was 15. The 10th percentile, median, and 90th percentile of the slice spacing were 2.75, 3.00, and 3.60 mm, and those of the in-plane spacing were 0.42, 0.59, and 0.80 mm, respectively.

Among the subjects for whom we know the MS type, 70% were diagnosed with relapse-remitting MS, 18% had a progressive disease course, 3% had clinically isolated syndrome, and 9% of subjects in the dataset were healthy control subjects.

**Fig. 1.**
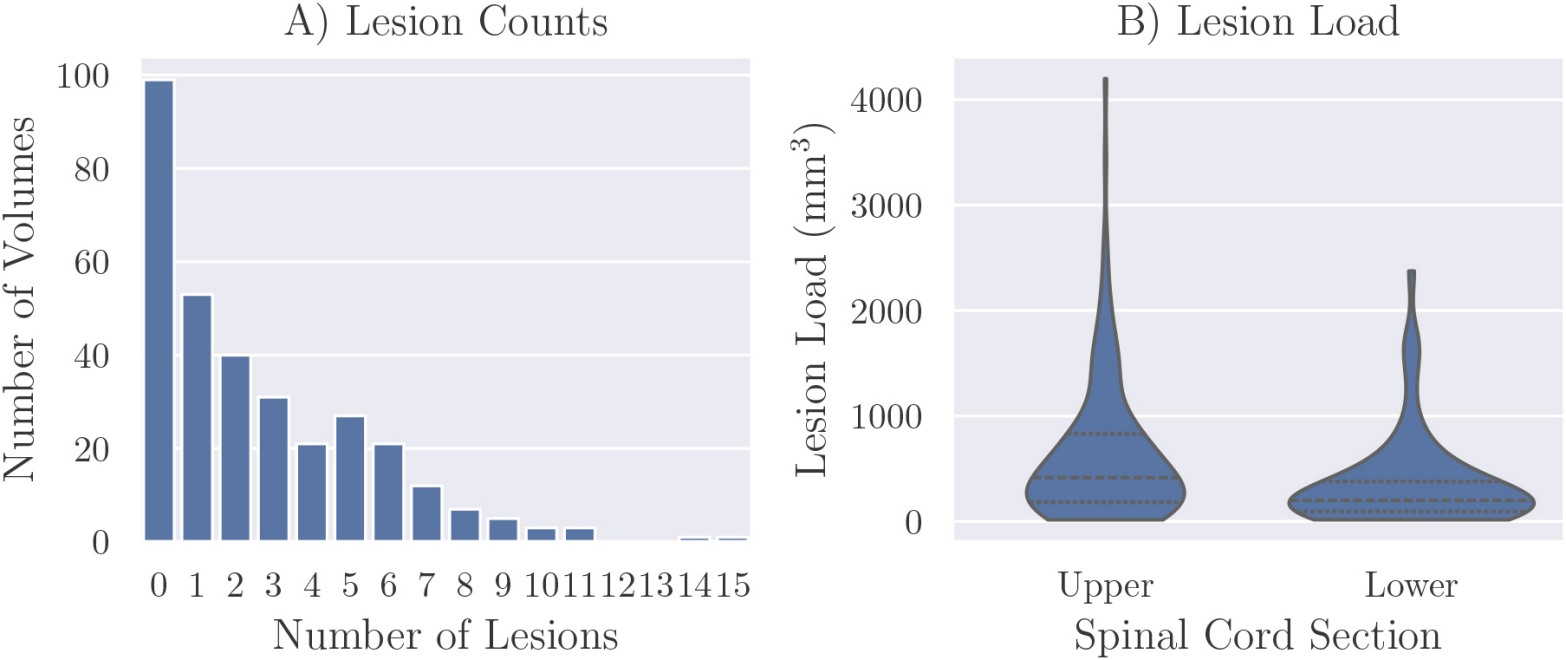
Lesion counts (A) and distribution of lesion load (B) per MR volume across the whole dataset. The lesion load distribution shown includes only volumes containing at least one lesion. The dashed lines represent the 1st quartile, median, and 3rd quartile.

Ninety-nine of the 324 volumes had no lesions present, with 26 of these coming from healthy control subjects. Among the 225 volumes with lesions, the median number of lesions was 3. The full distribution of lesion counts is shown in Figure 1A. Lesion load, similarly to lesion count, had a skewed-right distribution with a range of [15, 4200] mm^3^, median lesion load of 332 mm^3^, and first and third quartiles of 146 mm^3^ and 705 mm^3^, respectively. Figure 1B shows that this skewed distribution is present in both upper and lower acquisitions.

To create manual annotations for the 324 volumes, each sagittal T2-w acquisition was reviewed by one of six expert neuroradiologists and neurologists. These experts also had access to supplementary acquisitions (e.g., STIR, axial T2-w, or axial $\text{T}2^{*}$-w) for each subject. Raters were instructed to segment only the hyperintense area, and to only segment lesions below the pons. Rater instructions did not indicate a minimum lesion size. The segmentation masks created by these experts were then validated and adjusted where necessary by a senior neurologist with 10 years’ experience in reading spinal cord MRI. A segmented area was accepted only if there was a high confidence of it being a lesion (e.g., appearing on multiple scans). To aid the annotation process and reduce the chance of missed lesions, a deep learning model was trained on a subset of 62 volumes, as detailed in Walsh et al. (2023), and was used to obtain predicted segmentations for all 324 volumes (including those used for training). The predicted segmentations were reviewed by the senior expert, who decided whether to include or reject the proposed lesions, and the segmentations were manually corrected if necessary.

### 2.2 Training and test sets

The data splits were chosen to be consistent with previous projects on the same data (e.g., Lodé et al., 2025), where a subset of 217 of the 324 volumes was split into folds for 5-fold cross-validation. Three folds were used for training in each run, one fold was used as a validation set, and the remaining fold functioned as a held-out test fold. The splits were stratified on scanner vendor and lesion load, ensuring acquisitions for a single subject were only in one fold. Experiments were conducted with different training set sizes (see Section 4.2); for the largest training set size, all remaining volumes in the dataset not contained in the subset of 217 volumes were included.

To supplement the 40–50 volumes in the test folds, and to increase the statistical confidence of our results, we also used an additional dataset created by Lodé et al. (2025). This included 100 sagittal T2-w volumes (50 upper, 50 lower) obtained from OFSEP. The final test set used for each run of each experiment thus contained 140–150 volumes.

The extra 100 volumes from Lodé et al. (2025) were collected and annotated independently from the 324 volumes used in this study, and were never used for training or analysis—they were only used for testing. As described in Lodé et al. (2025), the annotations for these 100 volumes were created with the input of 20 experts with a range of experience. Each expert reviewed each acquisition together with a corresponding STIR acquisition in two sessions, with and without the aid of an automated segmentation tool, and clicked areas of the acquisition where they suspected lesions. Other than the fact that these annotations were created by single clicks on a lesion rather than full segmentations, the rest of the annotation instructions were similar to those used for the 324 volumes described above. The final segmentation masks for these 100 volumes were created by a radiology resident and validated by a neurologist with 10 years’ experience. To create these masks, the annotators reviewed the T2-w and STIR volumes, the inputs of the 20 experts and automated segmentation tool, and supplementary acquisitions for the patient (such as axial T2-w or $\text{T}2^{*}$-w), where available. Discrepancies were adjudicated by a third expert, a neuroradiologist with 6 years’ experience.

### 2.3 Preprocessing

The volumes were preprocessed in a similar way to previous studies (Gros et al., 2019; Walsh et al., 2023, 2024), and full details for the preprocessing used in this study are given in the Supplementary Material. Briefly, volumes were resampled to 0.5 mm^3^ isotropic spacing. Each axial slice was cropped to (48, 48) voxels around the spinal cord with the aid of spinal cord segmentation masks obtained with the Spinal Cord Toolbox (SCT) (De Leener et al., 2017). These axial slices were stacked along the z-axis, leading to a “straightened” spinal cord representation. The resulting length of the preprocessed images along the z-axis ranged from 187 to 909, with a median of 478 voxels (239 mm).

## 3 Synthetic Lesion Generation (LesionSCynth)

**Fig. 2.**
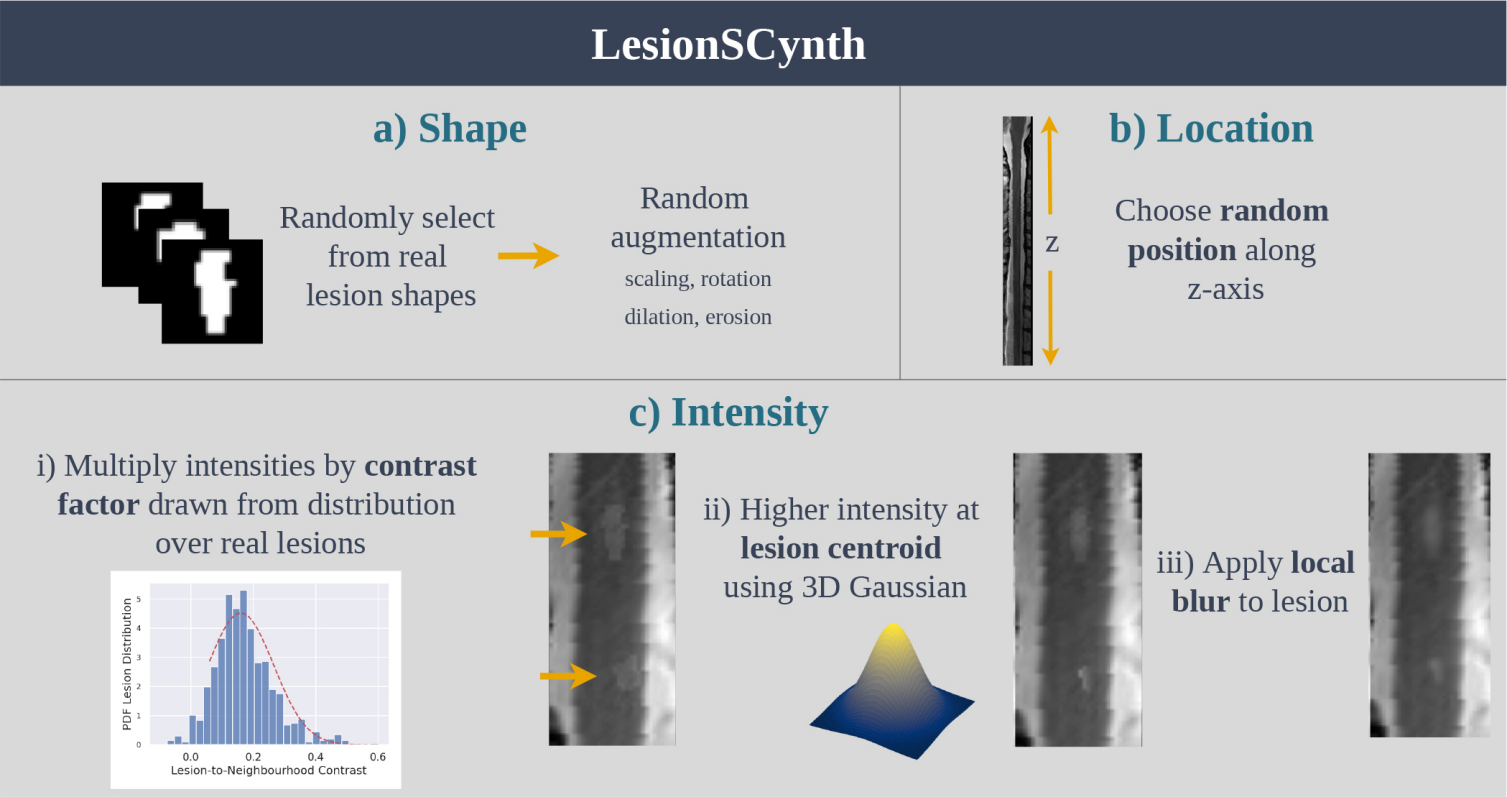
Overview of LesionSCynth for synthesising spinal cord lesions given a small set of real lesions.

We aim to build a simple parametric process to generate altered versions of the training data that includes prior knowledge of lesion appearance. This section provides a principled description of such a parametric generating process to add an arbitrary number of synthetic lesions to acquisitions using a limited number of lesions provided in the training set. The subsequent sections of the paper will describe how this process was used to increase the diversity of lesions when training a U-Net model. The overall process is illustrated in Figure 2 and detailed below.

### 3.1 Lesion intensity profiles

#### 3.1.1 Lesion-to-normal-appearing-tissue contrast

The main defining feature of MS lesion appearance is its contrast relative to surrounding tissue. Here, we describe a method to compute and analyse the contrast of a given lesion relative to its neighbouring normal-appearing tissue. For a lesion p, we have a manual binary segmentation mask $M_{p}$. To obtain the mask of the lesion neighbourhood on each slice, we first dilated $M_{p}$ in each sagittal slice using a disc structuring element with a radius of five voxels, and then subtracted the original $M_{p}$. We performed the dilation within sagittal slices rather than in 3D because the in-plane spacing is much smaller (approx 0.5 mm) than the between-slice spacing (approx 3 mm), which can cause abrupt intensity changes between consecutive slices. To prevent hyperintense CSF being included in the neighbourhood, we removed any voxels outside the spinal cord by multiplying the result with the spinal cord mask after applying an erosion using a disc of radius one voxel. The resulting neighbourhood mask for lesion p is denoted $M_{p}^{*}$. The contrast ratio, $C_{p}$, between the lesion and the surrounding normal tissue is calculated as:

$$C_{p}=\frac{\frac{1}{\left|M_{p}^{*}\right|}\sum_{\text{v}\in M_{p}^{*}}I_{\text{v}}-\frac{1}{\left|M_{p}\right|}\sum_{\text{v}\in M_{p}}I_{\text{v}}}{\frac{1}{\left|M_{p}^{*}\right|}\sum_{\text{v}\in M_{p}^{*}}I_{\text{v}}},$$ (1)

where $I_{\text{v}}$ is the intensity at some voxel v=(i,j,k) and $\left|M_{p}\right|$ is the number of voxels in mask $M_{p}$. We used the mean intensity for this calculation because it is the standard choice in MRI for intensity-based metrics such as signal-to-noise ratio and is commonly used to quantify lesion contrast relative to normal tissue (Mirafzal et al., 2020). We also confirmed that using the median yielded a similar approximately normal distribution of contrast values, indicating that the choice of mean does not substantially affect the resulting distribution from which contrast factors are sampled.

**Fig. 3.**
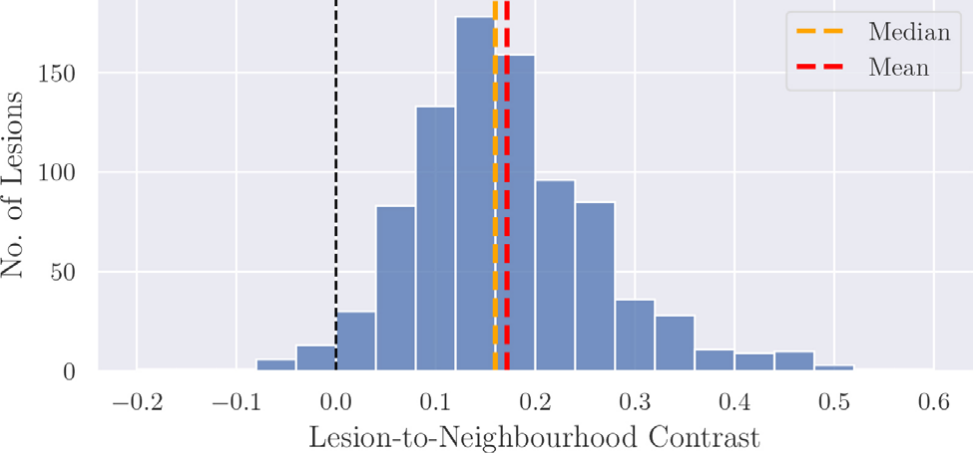
Distribution of contrast between mean lesion intensity and mean neighbourhood intensity. The sample mean is 0.17 and the sample standard deviation is 0.11 .

Figure 3 displays the distribution of C from the 847 lesions in the dataset. Overall, we observed that the contrast between mean lesion intensity and neighbourhood follows an approximate normal distribution. Lesions with low and negative contrast in Figure 3 typically occur when (1) the neighbourhood includes voxels with CSF partial volume effects, despite efforts to exclude them, (2) the lesion is not clearly distinguishable in the sagittal T2-w acquisition but appears more clearly in other acquisitions, or (3) the manual segmentation either misses some hyperintense regions or includes voxels with lower intensity. Examples are given in the Supplementary Material.

A simple approach to take into account this contrast distribution to synthesise a lesion would be to increase the intensity inside a candidate lesion region, $M_{s}$, such that the mean intensity is increased by a multiplicative factor, that is,

$$\frac{1}{\left|M_{s}\right|}\sum_{\text{v}\in M_{s}}I_{\text{v}}^{*}=\left(1+c\right)\times \frac{1}{\left|M_{s}\right|}\sum_{\text{v}\in M_{s}}I_{\text{v}},$$ (2)

for example, by increasing all intensities by this multiplicative factor inside the candidate lesion region, that is, $I_{\text{v}}^{*}:=(1+c)\times I_{\text{v}},\forall \text{v}\in M_{s}$, where I is the original intensity of normal-appearing tissue, $I^{*}$ is the updated intensity, and c is a contrast factor sampled from a distribution ψ, chosen to follow that of the empirical distribution in Figure 3.

In our experiments, we modelled c as a truncated normal distribution $\psi \left(\bar{\mu },\bar{\sigma },a,b\right)$, with the parameters computed from the available lesions in the training set of a given experiment. First, the contrast was calculated between the mean intensity of each lesion and its surrounding tissue. $\bar{\mu },\bar{\sigma },a$ were then given by the mean, standard deviation, and 20th percentile over these values, respectively. b was set to 1.0 in our experiments, that is, the right side of the distribution was effectively not truncated. The left side was truncated at the 20th percentile of real cases to avoid sampling very low or negative contrast values.

#### 3.1.2 Hyperintensity is more pronounced near lesion centre

**Fig. 4.**
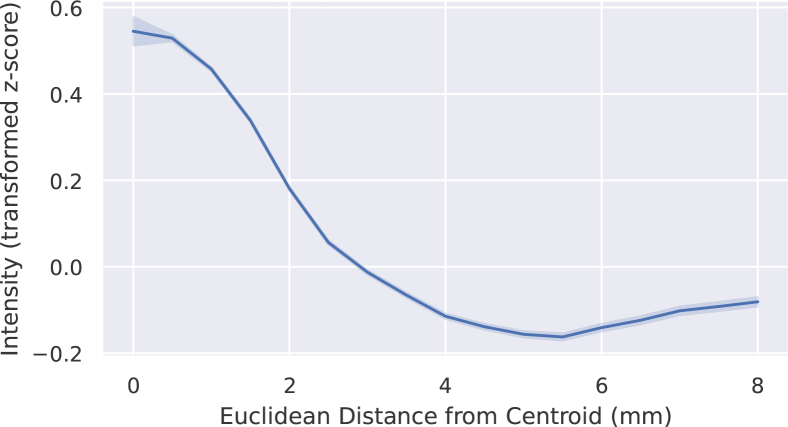
Mean intensity of lesion voxels as a function of the distance from the lesion centroid, based on all lesions in the dataset. Distances are rounded to the nearest 0.5 mm, and bounds are ±1.96×SE , where standard error is calculated across all voxels from all lesions. See Supplementary Material for further details on how this graph was generated.

Intensity in lesions is typically not spatially uniform. In particular, intensities tend to be higher near the lesion’s centre of mass and to decrease towards its boundary. This tendency is illustrated in Figure 4 (see Supplementary Material for details on this analysis).

This observation, together with the intensity scaling defined in Eq. (2), motivated the following rule to model lesion hyperintensity at each voxel $\left(i,j,k\right)\in M_{c}$:

$$I_{i,j,k}^{*}=I_{i,j,k}\times \left(1+c\times f_{M_{c}}\left(\left|i-i_{0}\right|,\left|j-j_{0}\right|,\left|k-k_{0}\right|\right)\right),$$ (3)

where

- $f_{M_{c}}\;{ℛ}_{+}^{3}\to {ℛ}_{+}$ is any decreasing function satisfying $\sum_{(i,j,k)\in M_{c}}f\left(\left|i-i_{0}\right|,\;\left|j-j_{0}\right|,\;\left|k-k_{0}\right|\right)=1$ ,
- $\left(i_{0},j_{0},k_{0}\right)$ is the coordinate of the lesion’s centre of mass.

In practice, we used a 3D Gaussian function whose scale parameters were independently fitted to the lesion’s spatial extent in each dimension:

$$I_{i,j,k}^{*}=I_{i,j,k}\times \left(1+Ae^{-\left(\frac{{\left(i-i_{0}\right)}^{2}}{2\sigma_{I}^{2}}+\frac{{\left(j-j_{0}\right)}^{2}}{2\sigma_{J}^{2}}+\frac{{\left(k-k_{0}\right)}^{2}}{2\sigma_{K}^{2}}\right)}\right),$$ (4)

where

$$A=\frac{c\left|M_{c}\right|}{\sum_{\left(i,j,k\right)\in M_{c}}e^{-\left(\frac{{\left(i-i_{0}\right)}^{2}}{2\sigma_{I}^{2}}+\frac{{\left(j-j_{0}\right)}^{2}}{2\sigma_{J}^{2}}+\frac{{\left(k-k_{0}\right)}^{2}}{2\sigma_{K}^{2}}\right)}}.$$

Parameters $\sigma_{I},\sigma_{J},\;\text{and}\;\sigma_{K}$ control how quickly the intensity increase decays along each axis. These rates were dynamically adjusted based on both the size and shape of the lesion. The decay of the Gaussian function, determined by $\sigma_{I}$, $\sigma_{J}$, and $\sigma_{K}$, should be related to the length of the lesion. For example, a short lesion requires a sharper decay, that is, a lower σ; if not, there would be very little difference across the whole lesion. However, a long lesion requires a slower decay, that is, a higher σ; otherwise, the function would quickly taper to 0 and most of the lesion would receive no intensity increase. Similarly, σ should be independently determined for each axis, given that the length of the lesion along each axis can vary significantly. For a given lesion with length along axis m given by $l_{m}$, we sampled $\sigma_{m}∼U(l_{m}/8,l_{m})$ . To avoid cases where part of the lesion area receives no intensity increase (typically when c is small and $\sigma_{m}$ is close to $l_{m}/8$ ), we applied a minimum intensity increase of 0.015 to ensure at least some small signal is present within the entire candidate lesion mask. Overall, the process described here allows diversity in the hyperintensity profile, ranging from a very sharp decay when $\sigma_{m}\approx l_{m}/8$ to virtually no spatial gradient when $\sigma_{m}\approx \;\;l_{m}$.

It should be noted that the generated intensity profile is not strictly decreasing from the centre of mass as it incorporates variation from the normal-appearing spinal cord in the target image.

#### 3.1.3 Intensity homogeneity

**Fig. 5.**
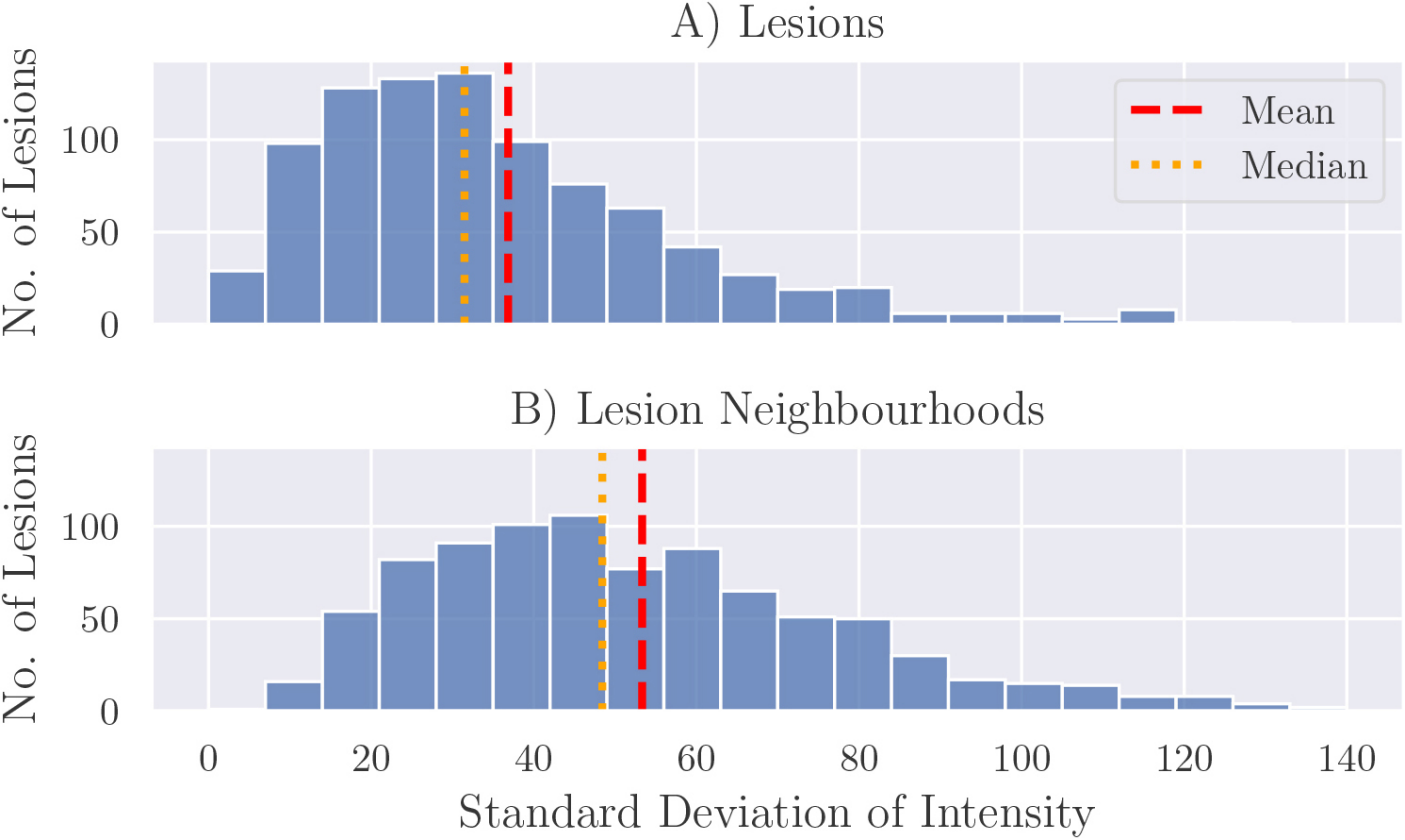
Distributions of standard deviation of intensities within (A) lesions and (B) neighbourhoods around lesions, with the latter obtained by dilating the lesion mask with a disc structuring element of radius five voxels and masking with the spinal cord segmentation mask.

As illustrated in Figure 5, variance of intensity tends to be lower within lesions than within lesion neighbourhoods. The final step in generating our synthetic lesions intensity profile is thus to apply a blur filter to the lesion to increase local homogeneity and to reduce the intensity variance inside the lesion. This is applied with the *gaussian_filter* function from the Scipy package, with blur radius set to 2 and blur sigma set to 0.67. These values were chosen after visually inspecting how realistic generated examples were.

**Fig. 6.**
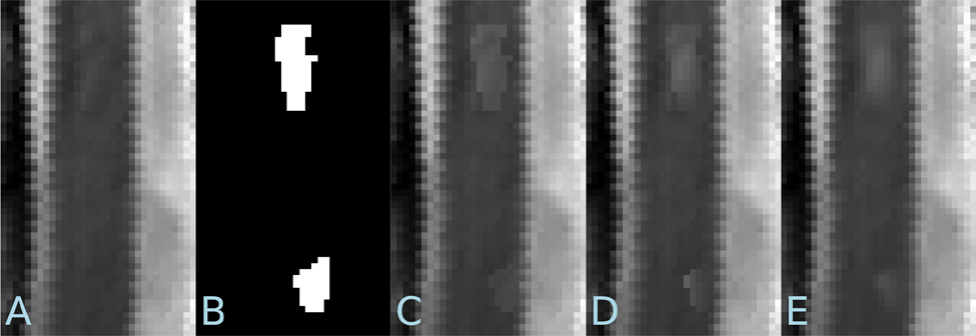
Examples of synthetic lesions. (A) Sagittal cross-section of image with no real lesions (patch size: 16×27.5 mm). (B) Masks of two lesions to be inserted. (C) Result of multiplying intensity of all voxels inside masks by a factor of 1.2. (D) Application of spatial Gaussian function (σ=1×2.5 mm) to the intensity increase. (E) Application of blur to the output of (D) with radius = 1 mm and σ=0.333 mm.

Figure 6 illustrates examples of the appearance of synthetic lesions after each of the three steps in the process to generate the intensity profile. Simply increasing the intensity in the candidate lesion leads to a clear step change in intensity that is unrealistic in real lesions (Fig. 6C). The subsequent two steps of the process mitigate this problem and create lesions that appear more realistic. However, as shown in the ablation studies in Section 5.4, these additional two steps are not strictly necessary to obtain the benefit to a deep learning segmentation model when training with synthetic lesions alongside a small set of real lesions.

### 3.2 Lesion shapes and locations

The previous sections described the process to generate lesion intensity profile by assuming that a mask $M_{c}$ for a candidate lesion is available. In this section, we describe how this mask is created. This process takes place after the data preprocessing outlined in Section 2.3 and described in detail in the Supplementary Material. In particular, in this section, we assume that images have 0.5 mm isotropic spacing and have been cropped and centred around the spinal cord.

#### 3.2.1 Lesion shape and orientation

Lesions vary in size and shape. In this work, we used a very simple approach with the lesion masks from the training set annotations. In practice, a lesion from the training set was first randomly selected from lesions with volumes between the 10th and 90th percentiles, to avoid extremely small or large lesions. Then, two steps were applied to introduce slight deviations from the original lesion mask. First, a random scaling and rotation operation was applied to the lesion mask with probability p=0.5 . The scaling factor was sampled uniformly from [0.9,1.1] and rotation degrees were sampled independently for each axis from (U(−5,5),U(−5,5),U(−45,45) ) (in LAS+ orientation). Second, the mask was either eroded (p=0.25 ) or dilated (p=0.25 ) using a ball structuring element with radius one voxel.

These operations introduced variation in lesion shape and size, while mostly maintaining clinical plausibility, as most transformations are limited to slight variations. However, rotating around the S-I axis by up to 45 degrees can occasionally place lesions in locations that are unlikely in real cases. This represents a trade-off between increasing variability in lesion shapes and orientations, and ensuring that all generated lesions remain anatomically plausible. Incorporating anatomical priors could help address this in future work.

#### 3.2.2 Lesion position

Given a target MR volume, the centroid of the candidate lesion was placed at a random point along the z-axis. The axial position of the source lesion was used, though altered by the rotation operation described above. No registration was needed as volumes were roughly aligned axially after preprocessing. Prior probability maps may also be used to choose lesion position, as in LesionMix (Basaran et al., 2024), but in cases with few annotated examples, this may not be appropriate as the full range of lesion positions would not be represented. After placing the source lesion mask in the target image, any area outside the spinal cord was removed. This was done using the spinal cord mask, after eroding with a 1-voxel radius disc structuring element to reduce the chance of CSF voxels being included in synthetic lesions.

### 3.3 Summary of the lesion synthesis process

#### 3.3.1 Capturing parameters

Parameters were computed from the set of lesions in the training set (which changed from one experiment to another). To avoid data leakage, the validation and test sets were not used to compute parameters.

1. Compute the mean and standard deviation of the contrast between the lesions and their surrounding normal-appearing tissue. These parametrise the contrast distribution used for synthesis.
2. Extract the segmentation masks, to be used as the lesion shapes in the synthesised examples.

#### 3.3.2 Producing a synthetic case

For a given acquisition containing no lesions:

1. Choose a random position and insert a random segmentation mask from the training set, after applying some augmentation transformations to the mask.
2. Synthesise the appearance of a lesion within the candidate mask by increasing the mean intensity by a multiplicative factor, inducing a spatial gradient in the intensity increase using a Gaussian function, and increasing local homogeneity of the intensity with a blur applied locally.

## 4 Experiments

We evaluated the impact of our synthetic lesion generation framework through a series of controlled experiments. Specifically, we assessed segmentation and detection performance when training with a combination of LesionSCynth and real lesions, and we compared with two synthesis alternatives and training on real data alone. All of these experiments were conducted at four different training set sizes. Finally, we examined several components of the method to understand the contribution of each part. The experimental settings are described in more detail below.

### 4.1 Training strategies

We trained models using different combinations of real and synthetic lesion data to isolate the contribution of each. All experiments share the same architecture and training hyperparameters, which will be described in Section 4.4.

#### 4.1.1 LesionSCynth

In our base training setup, each epoch included all real volumes containing ground-truth (GT) lesions, along with an equal number of synthetic examples. These synthetic examples were created by inserting lesions into real volumes without GT lesions, similar to the training process from Walsh and Tardy (2023). The number of synthetic lesions inserted per volume was a random whole number between 0 and 8, inspired by the finding by Basaran et al. (2024) that using a uniform distribution for target lesion load achieved the best performance. Only lesions in the training set for a given experiment were used to compute and initialise the parameters for LesionSCynth; validation and test data were never used for parameter estimation.

It is unclear a priori whether real or synthetic lesions should be seen relatively more frequently during training. On the one hand, we want the segmentation model to learn the characteristics of real lesions, but on the other hand, if few real lesions are available, inserting a higher proportion of synthetic lesions may help reduce overfitting. Thus, while our base method used 50% real and 50% synthetic examples per epoch, we also examined scenarios with 25%/75% and 75%/25% real and synthetic examples (the number of iterations was consistent with the base 50/50 experiments). The results of these extra experiments are presented in Section 5.4.

#### 4.1.2 Synthetic lesion baselines

To contextualise our method, we compared it with two state-of-the-art lesion synthesis methods, CarveMix and LesionMix. For both synthesis baselines, we followed the same training framework as for our method, that is, the only difference with the training method described in Section 4.1.1 is that the synthetic examples were created with an alternative method.

##### 4.1.2.1 CarveMix

The original implementation of CarveMix involved generating a fixed number of synthetic examples offline before training, and so we re-implemented this as a TorchIO transform that can be used on-the-fly during training. Moreover, CarveMix was originally proposed as a general data augmentation technique, that is, to mix any pair of images in the training set. Therefore, our use of CarveMix to only insert real lesions into an image with no lesions deviates from the original proposed usage. However, we aim to isolate the impact of the lesion synthesis part and so prefer to align with the training framework of our proposed method.

The intensity profiles varied substantially among the images in our training set. Therefore, a local hyperintensity in one image may be a hypointensity when that region is copied into another image. Standardising both images to have mean 0 and standard deviation 1, as suggested by X. Zhang et al. (2023), helped to reduce the impact of this, but did not fully eliminate this problem. Examples of lesions generated with CarveMix are given in the Supplementary Material.

##### 4.1.2.2 LesionMix

The original LesionMix contains two branches, one for *populating* an image with a new lesion and another for *removing* an existing lesion from an image using inpainting. Since, as before with CarveMix, we wish to assess only the effectiveness of the synthetic lesions, we use only this part of LesionMix, which we refer to hereafter as ${\text{LesionMix}}_{pop}$ .

Given that the code for LesionMix is not publicly available, we implemented a version of ${\text{LesionMix}}_{pop}$ as a TorchIO transform. First, before training, all lesions were extracted from the standardised training images in patches of size 48×48×b , where b is the length of the bounding box of the lesion along the z-axis. During training, lesions were randomly inserted into volumes without GT lesions such that the total lesion load reaches a target load. This target was sampled from a uniform distribution between the 5th and 95th percentiles of the lesion load in the training samples having real lesions, as Basaran et al. (2024) found that this uniform distribution yielded the best performance in their experiments.

As in the original paper, several augmentations were applied to the original lesions before being inserted into a new volume. In particular, random flips with p=0.5 for each axis, random scaling between 50% and 180% with p=0.5 , elastic deformation with maximum displacement of 4 voxels and p=0.5 , and gamma intensity transformation. Although Basaran et al. (2024) applied noise with σ=1 , this level of noise dominated the lesions in our case, so we reduced this to σ=0.1 . We applied random rotation around the z-axis with degrees ≤89 with p=0.5 as in the original paper, but we limited rotation around the other axes to [−5,5] degrees because our patches are narrow in the x-y plane.

We matched the choice of inserted lesion position with our method, that is, the position is randomly varied along the z-axis, and any area of the inserted lesion that lies outside the spinal cord mask was removed.

#### 4.1.3 Real-only baselines

To understand the contributions of synthetic data, we also examined models trained exclusively on real data under various sampling strategies.

When comparing with a real-image-only approach, two important questions arise: (1) could any observed benefit of our method be simply because of the introduction of a larger variability in normal anatomy and imaging characteristics and (2) is the benefit of the method due simply to a higher frequency of lesions observed during training, that is, because we reduce class imbalance.

##### 4.1.3.1 Real—balanced

To address the first question above, we take the same training framework as for our method, where the only difference is that no synthetic lesions are inserted. In other words, for each training epoch, the model will see all examples with real annotated lesions and an equal number of real volumes with no lesions. We assess whether there is an impact on the proportion of lesion/no lesion by also running experiments with 25%/75% and 75%/25% lesion/no lesion examples, respectively. The number of iterations and epochs was kept consistent with the base experiments.

##### 4.1.3.2 Real—lesions only

To address the second question above, that is, is there a benefit from our method simply because of the higher frequency of lesions during training, we train a model which sees only examples with real lesions during training. To maintain consistency in the number of iterations and epochs, we sample four patches from each volume for each epoch rather than two patches per volume for the other experiments.

### 4.2 Training set size

To examine the impact of training set size on our method and the baselines, we conducted all experiments at four different training set scales. The number of volumes with real lesions used for training at each scale was 17, 36, 72, and 145. The volumes at scale 17 were a subset of those at scale 36, and so on. For all scales, the number of available real volumes with no GT lesions was approximately 70. These volumes were randomly sampled during each epoch to match the number of volumes with real lesions at that scale. At the highest scale (i.e., 145), this sampling procedure leads to oversampling the volumes with no lesions at every epoch. At each scale, the validation set contained volumes with real lesions and was approximately one-third the size of the training set. The test sets remained the same across all training set scales. For experiments where we vary the ratios of real to synthetic lesion volumes or lesion to no lesion volumes, we kept the number of epochs and iterations consistent with the balanced 50%/50% setting. For example, at scale 17 with a 25%/75% real/synthetic ratio, at each epoch, we randomly sampled 8–9 volumes with real lesions and 25–26 volumes with no lesions, and synthetic lesions were inserted into these latter volumes.

### 4.3 Method component analysis

We conducted several experiments to determine the impact of certain design choices in our method and experimental setup, and the results of these are presented in Section 5.4. First, as has been already described, we conducted experiments with different proportions of synthetic to real examples while training with LesionSCynth. Second, we examined the impact of three parts of the intensity synthesis, namely (1) simply increasing the intensity by a multiplicative factor, (2) inducing a spatial gradient to the intensity increase using a Gaussian function, and (3) applying a local blur after introducing the Gaussian spatial gradient. Third, we explored the effect of generating diverse synthetic examples on-the-fly compared with a small fixed set of synthetic examples. In the experiments described thus far in the paper, our method used a set of approximately 70 volumes with no GT lesions into which synthetic lesions were inserted on-the-fly during training. Moreover, these volumes were sampled randomly *per epoch* to match the number of volumes with real lesions at that training set scale. We tested a scenario where a fixed subsample of these volumes is selected randomly *before training* and a fixed set of synthetic lesions are inserted into these, thereby reducing the variability of both lesion appearance and normal-appearing anatomy observed during training. We conducted this experiment at the two lower training set scales. At scale 17, we use 17 real volumes with real lesions and a fixed set of 17 volumes with inserted lesions, and at scale 36, we use 36 in each group.

We carried out two ablation studies at training set scale 17 to assess the impact of varying lesion position and varying lesion shape and intensity. First, to isolate the effect of lesion position or coverage, we ran an experiment with a fixed lesion shape and intensity profile, and only varied the lesion position (in the same way as for the full method, that is, randomising position along the z-axis). The lesion shape chosen for this experiment was the lesion with the median volume from a set of lesions in 107 volumes external to the training and test sets. The hyperintensity was created by multiplying the intensities within the inserted lesion area by a fixed factor (0.2). Second, we ran an experiment with, conversely, a fixed position, where all real lesion shapes in the training volumes were used and the intensity profile was varied with the same factor distribution as the full LesionSCynth method. For this experiment, the synthetic lesions were inserted at the same position as their position in the original volumes, thereby removing the potential benefit of increased lesion coverage.

Finally, to further assess how impactful lesion coverage was on the improvement in performance, we also broke down the improvement between using real data versus LesionSCynth by spinal cord section. The volumes were already split into upper and lower acquisitions, where the upper acquisitions covered the cervical cord and upper thoracic cord, and the lower acquisitions covered the lower thoracic and lumbar cord. We further split the upper volumes into approximate cervical and upper thoracic sections using a fixed point along the z-axis obtained as the average position of the end of the cervical cord in five volumes.

### 4.4 Segmentation model

All training procedures described above were implemented using a consistent segmentation model, described below.

#### 4.4.1 Architecture

The model architecture was based on a fully 3D U-Net from nnU-Net (Isensee et al., 2021), a state-of-the-art medical image segmentation framework. We used a residual encoder, shown to outperform the base nnU-Net in many tasks in the recent update (Isensee et al., 2024). We limited the number of levels in the U-Net to five (i.e., four strided convolution operations) after observing no benefit to extra levels in preliminary experiments. We further applied dropout with p=0.2 . Although this is usually not applied in nnU-Net, the current study uses small training set sizes and so we applied dropout to help limit the risk of overfitting. Finally, we used a combined dice and cross-entropy loss, the default proposed by nnU-Net.

At inference, the model produces a probability map via a softmax activation, assigning each voxel a value between 0 and 1 that reflects its likelihood of being part of a lesion. These scores were not immediately binarised; instead, predictions were evaluated at multiple thresholds to compute the FROC score, as described in Section 4.5.

#### 4.4.2 Training

Patch size was set to 48×48×320 and two patches were randomly sampled from each preprocessed volume at each epoch. The batch size was set to eight. The total number of epochs was set such that the validation loss for most experiments had converged at that point. The number of iterations required until convergence was longer in experiments with more training data, and so the number of epochs was set to 2000 for scale = 17, 1500 for scale = 36, 1250 for scale = 72, and 1000 for scale = 145. The number of iterations per epoch was kept consistent across experiments for the same training data scale and was based on observing two patches per volume with real lesions and two patches from an equal number of volumes with no lesions or with synthetic lesions inserted. For example, the number of iterations per epoch at scale = 17 was $\left\lceil \frac{17\times 2\times 2}{8}\right\rceil =9$ , where 8 is the batch size.

During training we applied the following data augmentations, similar to nnU-Net, but here using the TorchIO library (Pérez-García et al., 2021): gamma intensity transform, flipping, blurring, simulating a bias field, downsampling, noise, and an affine transformation including rotation and scaling. Given the long and narrow nature of our patches, rotation was applied only around the z-axis, with a maximum of 30 degrees.

We assessed two hyperparameter settings. The first included the default optimiser, scheduler, and normalisation from nnU-Net, that is, SGD, polynomial decay of the learning rate, and InstanceNorm. The second used the AdamW optimiser with a weight decay factor of 0.01 , no learning rate scheduler, and GroupNorm with 16 groups. We speculated that if there were certain filter channels in the network associated primarily to recognising lesions and there were no lesions in a given patch, InstanceNorm may destroy this information, whereas GroupNorm allows some extra degrees of freedom so that this problem could be reduced. We ran 5-fold cross-validation with these 2 hyperparameter settings at two scales, 17 and 36, training with our method, and also the *Real – Lesions Only* and *Real – Balanced* baselines. The AdamW+GroupNorm setting achieved the highest FROC scores on the validation sets in all of the above cases, so we used this setting for the remaining experiments. The full results on the test sets for these two hyperparameter settings are available in the Supplementary Material.

During training, we saved checkpoints for two epochs, the epoch with the best loss on the validation set and also the final epoch. When evaluating each experiment, we calculated the FROC metric on the validation set for both checkpoints and selected the one which achieved the maximal performance.

### 4.5 Evaluation

#### 4.5.1 FROC

We used the evaluation protocol proposed by the MS-Multi-Spine Challenge on MS spinal cord lesion detection, which is taking place at MICCAI 2025.^1^ The challenge uses the FROC metric, which puts more emphasis on lesion detection rather than segmentation, as this is currently more important in clinical practice. In particular, given a prediction mask where each lesion has a predicted score or probability, the sensitivity is calculated at five levels of False Positive lesions Per Image (FPPI), namely 0.25, 0.5, 1, 2, and 3, where a predicted lesion is considered a true positive if the intersection over union (IoU) with a GT lesion is above 0.2. The FROC score is then the average of these five sensitivity values. Evaluating methods in such a way gives a fairer comparison and a more complete picture than evaluating at a single threshold.

To calculate the FROC score from the output of a segmentation model, that is, where each voxel is associated with an output score, a typical approach is to first binarise the mask at some threshold, compute the connected components, and then aggregate (e.g., mean or max) the voxel scores to have one score for each connected component. We tested two values for the initial binarisation threshold, 0.01 and 0.5, and two aggregation approaches, mean and max. In early experiments, max aggregation nearly always yielded superior FROC scores, so we used only max aggregation for the results reported in the paper. The optimal initial binarisation threshold, however, depended on the model, so we evaluated at both 0.01 and 0.5 for all experiments, and chose the threshold which yielded the highest performance on the validation set. For each of the five runs of each experiment, we, therefore, had four possible sets of predictions—two choices of saved checkpoint from the training run (see Section 4.4) and two choices of binarisation threshold.

In general, this procedure to choose which checkpoint and binarisation threshold to use tended to yield improved results. However, for experiments with a small validation set, individual images have a larger impact on the optimal choice for the validation set, and so this might diverge from the optimal configuration for the test set. Therefore, alongside the FROC score on the test set using the “best” configuration for the validation set, we also report the maximum possible FROC on the test set out of the four possibilities (two checkpoints and two binarisation thresholds), which we refer to as ${\text{FROC}}_{max}$ . This allows us to separate the effect of whether the training process allows to learn a better separation of lesion and non-lesion from the effect of choosing the “best” configuration based on the validation set.

#### 4.5.2 Single-threshold metrics

We report lesion sensitivity, lesion precision, lesion F1, and voxel-wise Dice score at the threshold which yields an average of one false positive per image (FPPI). FPPI = 1 was chosen as it allowed a reasonable balance between sensitivity and precision. Additional results for lesion sensitivity and precision at a fixed binarisation threshold of 0.5 are provided in the Supplementary Material. To separate segmentation performance from detection performance, we report ${\text{Dice}}_{IoU>0}$ , that is, Dice score calculated on the subset of GT lesions and predicted lesions that have a non-zero overlap with each other.

#### 4.5.3 Comparison with other models

In Section 5.5, we compare the best models in the current study with open-source lesion segmentation models from SCT and with the results reported in a clinical evaluation study (Lodé et al., 2025). To be able to compare directly with the results reported for the automated tool in Lodé et al. (2025), we must use the same data and evaluation metrics as that study: a GT lesion is detected if there is a non-zero overlap with the prediction, that is, at least one voxel is captured, and a predicted lesion is a false positive if it does not overlap with any GT lesion. This diverges from the other definition used in this study, which is based on a GT lesion requiring IoU>0.2 with a predicted lesion to be considered a true positive. We compare with the performance of the automated tool alone from Lodé et al. (2025)—in that study, when given the predictions from that automated tool, the lesion sensitivity among 20 experts improved by 5 percentage points on average. We compare the automated tools across studies to infer potential clinical impact. However, since we did not conduct a new reader study with the models from the current work, clinical impact is not demonstrated in this study.

#### 4.5.4 Statistical tests

Finally, we carried out two types of statistical tests in the study. First, when comparing two sets of FROC results, where we had five FROC values arising from the five cross-validation runs, we conducted a two-sided pairwise t-test. Second, when comparing metrics calculated at the image level (sensitivity, precision, etc.), we calculated the mean result for each volume across the five runs, and then conducted a two-sided pairwise Wilcoxon signed-rank test. Many volumes had values of 1 or 0 for these image-level metrics, and so a parametric t-test was not appropriate in this case.

## 5 Results

We first consider the high-level results of all methods in a low-data scenario, that is, at the lowest scale of training set size in Section 5.1, before going into more detail in comparing LesionSCynth versus using real images only in Section 5.2, and versus other synthesis methods in Section 5.3. Finally, we present the results of ablation studies and other investigations in Section 5.4, and compare with other spinal cord lesion segmentation models in Section 5.5.

### 5.1 Overall performance in low-data setting

Table 1 presents the results of all methods when trained at the lowest training set scale, that is, with 17 volumes with real lesions. Our method led to a better FROC score in this low-data setting than all other baselines. Neither CarveMix nor ${\text{LesionMix}}_{pop}$ improved over training with only real data. Training only on synthetic lesions from our method and excluding the 17 volumes with real lesions led to a much lower performance. This suggests that the characteristics of generated lesions are still relatively distinct from real lesions, and the benefit only comes when real and synthetic lesions are combined to broaden the learned distribution of lesion characteristics. Finally, although some differences with our method were not statistically significant, notably when compared with *Real – Lesions Only*, it should be noted that this may simply be a question of statistical power, given that only five observations were used for the statistical test. Indeed, looking at statistical significance using metrics calculated at the image level in Section 5.2, most differences were statistically significant.

**Table 1.** Performance when training at the lowest training set scale, that is, 17 volumes with real lesions in the training set.

METHOD	FROC Score	${\text{FROC}}_{max}$
Real – Balanced (50%/50% Lesions/No Lesions)	0.365* ± 0.051	0.365* ± 0.052
Real – Lesions Only (100% Lesions)	0.439 ± 0.091	0.455 ± 0.061
CarveMix (50%/50% Real/Synthetic)	0.405 ± 0.029	0.408* ± 0.025
${\text{LesionMix}}_{\text{pop}}$ (50%/50% Real/Synthetic)	0.404 ± 0.022	0.432* ± 0.039
LesionSCynth (100% Synthetic)	0.224* ± 0.076	0.249* ± 0.081
LesionSCynth (50%/50% Real/Synthetic)	**0.460** ± 0.066	**0.523** ± 0.036

Mean ± standard deviation was taken over five runs. The proportion in brackets refers to the proportion of volumes in each epoch, that is, volumes with lesions versus volumes without lesions when training on real data only, or volumes with real lesions versus volumes with synthetic lesions for the lesion synthesis methods. The best result for each metric is highlighted in bold.

* Statistically significant difference versus LesionSCynth (50%/50%) at 0.05 significance level.

### 5.2 Comparison with real-only baselines

**Fig. 7.**
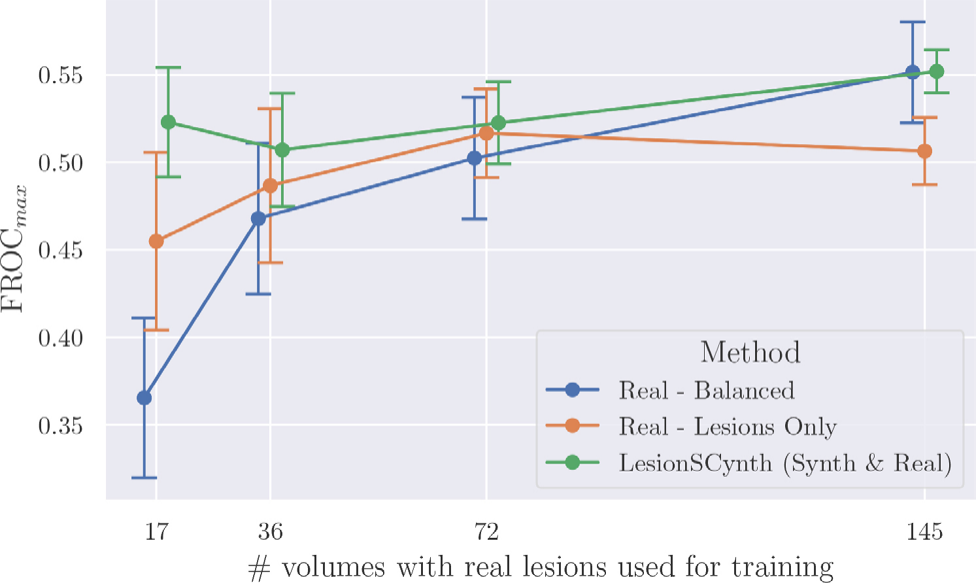
Performance at different training set scales for our method versus baselines using only real data. The best FROC score on the test folds is reported, with the mean and standard error (SE) calculated over five runs. Error bars are ±1.96×SE . Note that each of the methods was trained with the same amount of data, but the points are offset along the x-axis for clarity. Training set scales are 17, 36, 72, and 145 real volumes with lesions, with an equivalent number of volumes with no lesions or with synthetic lesions, depending on the method used.

Figure 7 presents the performance at different training set scales for our method versus baselines trained on real data only. As seen in the previous section, our method outperformed these baselines by a considerable margin at the lowest training set scale. However, this gap narrowed as the number of real volumes available for training increased. When 145 volumes with real lesions were available, then an equivalent performance was given by a baseline trained only on real data. Importantly, in all cases, our method did not deteriorate performance compared with training on real data only, and improved results when only a small number of annotated volumes were available.

As might be expected, the average performance increased in most cases when adding more training data. There were, however, 2 exceptions to this, including for LesionSCynth, where the FROC dropped by 0.016 between scales 17 and 36. Possible explanations for this are discussed in Section 6.

**Fig. 8.**
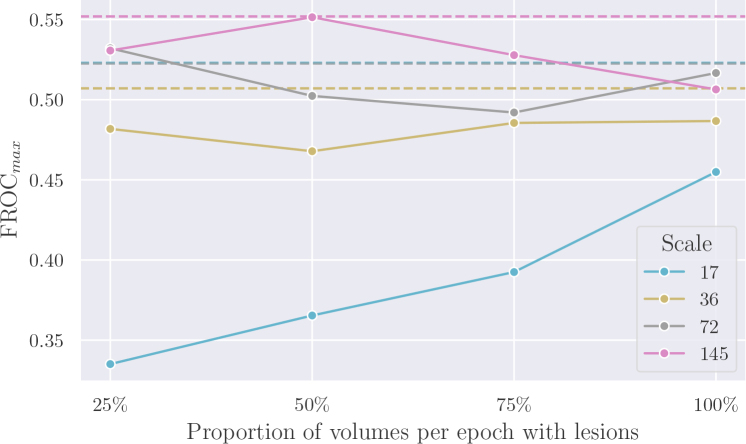
When using only real volumes during training, what is the effect of the proportion of volumes per epoch that contain lesions? The dashed lines represent the performance of our LesionSCynth method trained with real and synthetic lesions.

Figure 8 explores the relationship between performance and the proportion of volumes per epoch containing lesions when training only on real volumes. The point at 100% corresponds, therefore, to the *Real – Lesions Only* baseline in Figure 7 and 50% corresponds to the *Real – Balanced* baseline. The most striking pattern observed here is at training set scale 17, where performance increased significantly when using more volumes with lesions per epoch and fewer volumes with no lesions, with FROC increasing from 0.335 at 25% to 0.455 when only volumes with lesions were used for training. This pattern was not repeated at the other scales, and indeed no consistent pattern was discernible among the three larger training set sizes. When comparing each scale with the corresponding dashed line representing our method, we observed a clear increase in performance at scale 17 between the real baselines and our method. We note a more modest, but consistent, gap between the baselines and our method at scale 36, with FROC of 0.487 at 100% versus our method at 0.507. At the two highest training set scales, however, our method fell in a similar range to the baselines.

Moving from the multi-threshold evaluation given by FROC to an evaluation at individual thresholds on the predicted scores, Table 2 presents lesion-wise metrics alongside the Dice score for our method and the baselines on real data only. When evaluated at a point with an average of one false positive per image, our method consistently achieved the best lesion sensitivity and ${\text{Dice}}_{IoU>0}$ at all training set scales. However, the gap between our method and the next best result decreased as the training set scale increases, similarly to the previous observations on the FROC score. All experiments had relatively low Dice and lesion F1 scores, particularly if compared with brain lesion segmentation. However, Section 5.5 will put these results into a broader context and will show that these models are still clinically relevant.

**Table 2.** Evaluation at FPPI = 1 (mean ± std. dev. over five runs), that is, the softmax scores from each model were binarised at the threshold which yields one false positive per image on average on the test set (for that model).

		Lesion-wise Metrics			Voxel-wise Metrics	
Training Set Scale	METHOD	Sensitivity	Precision	F1	Dice	${\text{Dice}}_{IoU>0}$
17	Real – Lesions Only	0.42 ± $0.07^{**}$	0.45 ± $0.03^{**}$	0.36 ± $0.02^{**}$	0.31 ± $0.02^{**}$	0.51 ± $0.01^{**}$
	Real – Balanced	0.39 ± 0$.05^{**}$	0.56 ± $0.05^{**}$	0.41 ± $0.04^{**}$	0.30 ± $0.03^{**}$	0.51 $\pm \;\;0.02^{**}$
	LesionSCynth (Ours)	**0.52** ± 0.05	**0.58** ± 0.01	**0.45** ± 0.05	**0.37** ± 0.03	**0.55** ± 0.02
36	Real – Lesions Only	0.47 ± $0.06^{**}$	0.54 ± 0.04	0.42 ± $0.06^{**}$	0.34 ± $0.03^{**}$	0.54 ± 0.03
	Real – Balanced	0.50 ± $0.05^{**}$	**0.60** ± 0.04	**0.48** ± $0.05^{**}$	0.36 ± 0.02	0.55 ± 0.03
	LesionSCynth (Ours)	**0.52** ± 0.03	0.55 ± 0.02	0.45 ± 0.03	**0.37** ± 0.02	**0.56** ± 0.02
72	Real – Lesions Only	0.53 ± 0.04	0.53 ± $0.04^{*}$	0.44 ± 0.05	**0.40** ± 0.02	**0.58** ± 0.02
	Real – Balanced	0.53 ± 0.04	**0.56** ± 0.01	**0.46** ± 0.03	0.37 ± 0.02	0.55 $\pm \;\;0.02^{**}$
	LesionSCynth (Ours)	**0.54** ± 0.03	0.55 ± 0.03	**0.46** ± 0.04	0.39 ± 0.03	**0.58** ± 0.004
145	Real – Lesions Only	0.53 ± $0.02^{**}$	0.52 ± 0.03	0.42 ± $0.03^{*}$	0.39 ± 0.02	0.58 ± 0.02
	Real – Balanced	**0.59** ± 0.03	**0.57** ± 0.02	**0.49** ± 0.02	**0.41** ± 0.03	0.58 ± 0.01
	LesionSCynth (Ours)	**0.59** ± 0.01	0.56 ± 0.02	0.48 ± 0.02	0.40 ± 0.03	**0.59** ± 0.01

Lesion sensitivity, lesion precision, and lesion F1 score are reported, alongside Dice score and Dice evaluated only on GT and predicted lesions with non-zero overlap (${\text{Dice}}_{IoU>0}$). **Real – Lesions Only**: Baseline trained on 100% real volumes with lesions; **Real – Balanced**: Baseline trained on real volumes, balanced per epoch between volumes with real lesions and volumes with no lesions (50% volumes with lesions/50% volumes with no lesions per epoch); **LesionSCynth**: trained on a mix of volumes with real lesions and synthetic lesions (50% volumes with real lesions/50% volumes with synthetic lesions per epoch). The best result at each training set scale is highlighted in bold.

**: 0.01; *: 0.05—statistically significant difference, comparing with LesionSCynth at the same scale.

**Fig. 9.**
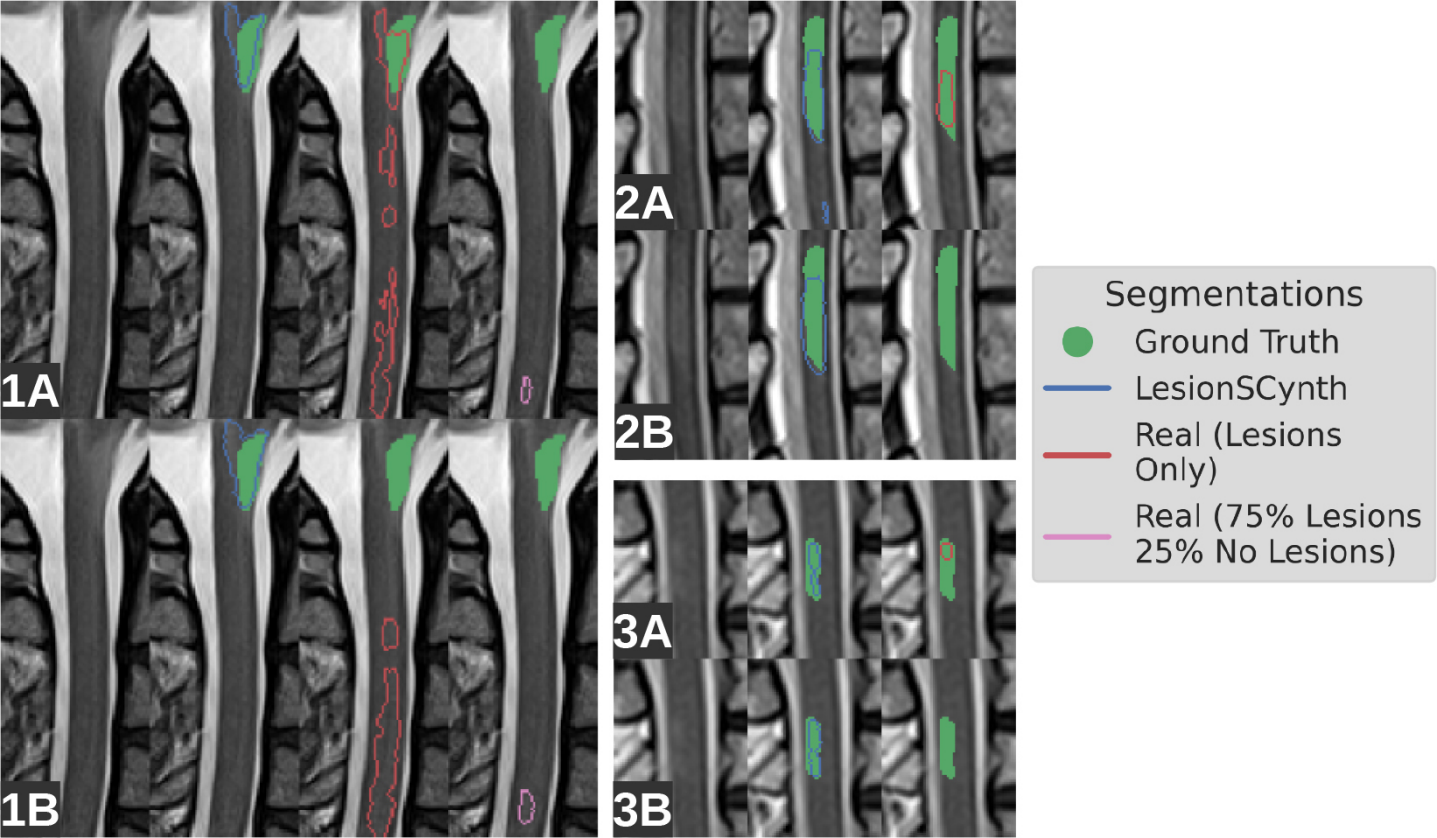
Examples (1-3) where adding synthetic lesions during training improves sensitivity. The first row (A) in each plot is the result from a simple threshold of 0.5 on the output softmax scores, whereas the second row (B) involves first thresholding at 0.01, computing connected components, and applying a second threshold which achieves an average of one false positive per image. See the description of FROC for more details on this process (Section 4.5).

Finally, Figure 9 shows the segmentations for three lesion examples in the dataset where training with LesionSCynth improves performance compared with real-only baselines. *Real – Lesions Only* model did segment at least part of these three lesions using a threshold of 0.5 on the output scores. However, the outputs for this model also contained many false positives, so applying a threshold to give one false positive per image on average led to the true positive predictions being removed. For LesionSCynth, however, the true positive segmentations remained and a false positive in Figure 9-2A was removed when applying the threshold which yields FPPI = 1 (2B). Finally, none of these lesions were detected by the model trained only on real examples with 75% lesion examples and 25% non-lesion examples per epoch. The model segmented small parts of the lesions at thresh = $10^{-5}$ , but also segmented many false positives at this level, including even areas outside the spinal cord.

### 5.3 Comparison with synthetic lesion baselines

**Fig. 10.**
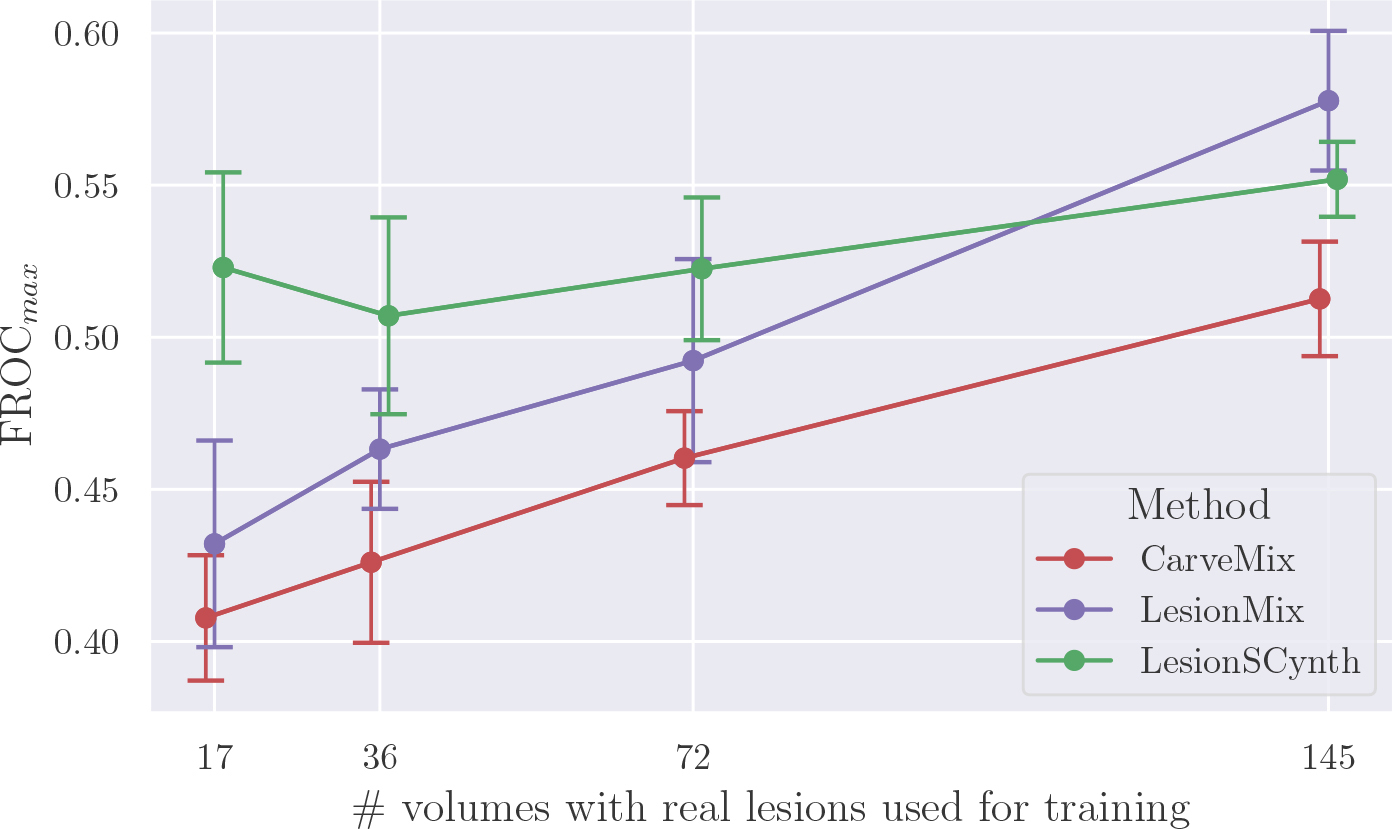
Performance at different training set scales for our method versus two lesion synthesis baselines, CarveMix and ${\text{LesionMix}}_{pop}$ . The best FROC score on the test folds is reported, with the mean and standard error (SE) calculated over five runs. Error bars are  ±1.96×SE . Note that each of the methods was trained with the same amount of data, but the points are offset along the x-axis for clarity. Training set scales are 17, 36, 72, and 145 real volumes with lesions, with an equivalent number of volumes with no lesions per epoch, into which synthetic lesions have been inserted using the respective synthesis method.

Similar to the trends observed when comparing with real-only baselines, we can see from Figure 10 that our method outperformed the state-of-the-art synthesis baselines by a large margin at small training set sizes, but this gap reduced as the training set size increased. Specifically, our method achieved the highest FROC scores at scales 17, 36, and 72. However, the FROC score for ${\text{LesionMix}}_{pop}$ increased impressively from 0.492 at scale 72 to 0.578 at scale 145, achieving the highest result at this scale, and indeed the highest result across all experiments and methods in this study. This trend is not surprising as when there are few volumes with real lesions available, synthesis methods based on directly copying existing lesions may overfit to the specific intensity patterns of this small set. In contrast, our parametric synthesis method can help the model to generalise to a wider distribution. However, given a large and diverse set of lesions in the training set, it can be more beneficial to use these as they can be more realistic than those synthesised by a parametric method. We note that CarveMix had the worst performance across all scales, indicating that it is not well adapted to the spinal cord lesion setting.

### 5.4 Method component analysis

#### 5.4.1 Synthetic lesion ratio

**Fig. 11.**
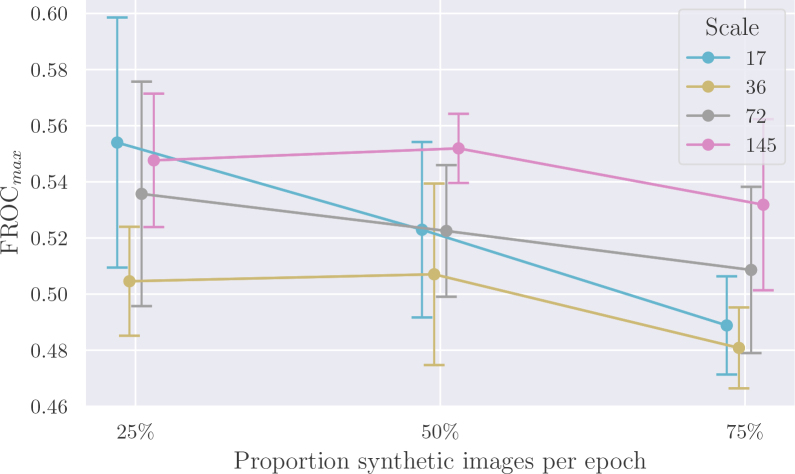
Effect of the proportion of volumes with synthetic lesions per epoch when training our method. For example, training with 25% synthetic examples means that for each epoch, there is a 3:1 ratio between real volumes with real lesions and volumes with inserted synthetic lesions. The best FROC score on the test folds is reported, with the mean and standard error (SE) calculated over five runs. Error bars are  ±1.96×SE .

Figure 11 examines the impact of different ratios of synthetic versus real lesion examples used per epoch. We note that there is a significant overlap in the confidence intervals for most of the observations, due in part to the fact that we have only five observations to calculate the standard error. However, even if the comparisons may not be statistically significant, the results still provide valuable insights. For example, the highest FROC score (${\text{FROC}}_{max}=0.554$ ) was observed at training scale 17 when 25% of volumes per epoch contained synthetic lesions and 75% were real. Indeed, this also outperformed all baselines trained on real volumes only. The standard error for this result is relatively large, influenced in particular by one outlier with a low value among the five runs. The FROC values for the five runs were 0.469 , 0.558 , 0.562 , 0.577 , and 0.603 . Conversely, the worst performance across scales occurred when 75% of volumes per epoch contained synthetic lesions and 25% were real.

#### 5.4.2 Ablation – lesion generation

Table 3 presents the results of ablation experiments on key parts of the synthesis process at the training set scale of 17 volumes with real lesions. In fact, there was very little change in the results between these three experiments, indicating that although the extra steps of introducing a spatial gradient and a local blur may increase how realistic the synthesised lesions are, these steps did not help the segmentation model to generalise further. Indeed, it seems sufficient to simply increase the intensity inside a candidate lesion area by a well-chosen multiplicative factor.

**Table 3.** Results without selected parts of synthesis method, at training set scale 17.

Gaussian Spatial Gradient	Blur	${\text{FROC}}_{max}$
		0.525 ± 0.057
✓		0.532 ± 0.040
✓	✓	0.523 ± 0.036

The differences were not statistically significant at the 0.05 significance level.

#### 5.4.3 On-the-fly versus fixed synthetic examples

Table 4 presents the results of using a fixed set of synthetic examples generated offline rather than generating more diverse examples on-the-fly during training. We can see that at scale 17, the standard setting with more diversity achieved a higher FROC with a difference of 0.022, whereas at scale 36 it is the opposite, where the setting with a fixed set of synthetic examples had a slightly higher performance with a difference of 0.009. In both cases, the differences were not statistically significant.

**Table 4.** Results when using a smaller fixed set of examples synthesised offline versus the default setting of creating more diverse synthetic examples on-the-fly while training our method.

Training set scale	Setting	${\text{FROC}}_{max}$
17	Synthesise on-the-fly (default)	**0.523** ± 0.036
	Fixed synthetic images	0.507 ± 0.070
36	Synthesise on-the-fly (default)	0.502 ± 0.062
	Fixed synthetic images	**0.511** ± 0.051

The differences were not statistically significant at the 0.05 significance level. The best result at each training set scale is highlighted in bold.

#### 5.4.4 Effect of coverage versus lesion diversity

Table 5 presents results from ablation studies to assess the impact of varying position, shape, and intensity when creating synthetic lesions. Varying only the synthetic lesion position but keeping a consistent lesion shape and intensity profile leads to a significant drop in performance compared with the full synthesis method, dropping from a FROC of 0.523 to 0.434. In contrast, using all available real lesion shapes in the training set, maintaining their original positions when inserting into new volumes, and varying only the intensity profile also fail to achieve the same performance as the full method (0.482 vs. 0.523, respectively). These results suggest that both parts of the synthesis pipeline are necessary and neither part is sufficient on its own to achieve a significant increase in performance.

**Table 5.** Ablation results for lesion position and lesion profile variation at training set scale 17.

METHOD	Multiple Lesion Shapes	Shape Augmentations	Varying Intensity	Varying Position	${\text{FROC}}_{max}$
LesionSCynth (50%/50%)				✓	0.434* ± 0.067
LesionSCynth (50%/50%)	✓		✓		0.482 ± 0.060
LesionSCynth (50%/50%)	✓	✓	✓	✓	0.523 ± 0.036

* : 0.05—statistically significant difference compared with full LesionSCynth (50%/50%) method.

Table 6 analyses the potential impact of improved lesion coverage from a different angle, by breaking down the difference in performance by spinal cord section. If the only mechanism by which the synthesis method helps is by increasing lesion coverage in under-represented areas (particularly the thoracic or lumbar cord sections), then we would expect to see less of an improvement at the cervical level when comparing LesionSCynth with training only on real data. However, the increase in performance with LesionSCynth is evident across all three spinal cord sections analysed here, with an increase in FROC of 0.095, 0.133, and 0.073 for the cervical, upper thoracic, and lower cord sections, respectively.

**Table 6.** Comparison of FROC score across different parts of the spinal cord at training set scale 17.

METHOD	Cerv	Upper Thor	Lower	All
Real – Lesions Only (100% Lesions)	0.500	0.450	0.484	0.455
LesionSCynth (75%/25% Real/Synthetic)	0.595*	0.583*	0.557	0.554*

“Lower” includes the lower thoracic cord and lumbar cord.

* : 0.05—statistically significant difference compared with *Real – Lesions Only*.

### 5.5 Comparison with other lesion segmentation tools

Finally, we place the models trained in this study in a broader context by comparing with existing open-source models from SCT (Benveniste et al., 2025; Gros et al., 2019) and with a model which helped to improve the lesion sensitivity of radiologists in a clinical evaluation study (Lodé et al., 2025). The results are shown in Table 7. We compare with the reported results for the automated tool in Lodé et al. (2025), but no new reader study was conducted to directly assess clinical impact of the models in the current study. Note that the definitions of lesion sensitivity and precision here are different than those used in the previous sections, in order to be able to compare with the results from Lodé et al. (2025)—see Section 4.5 for details.

**Table 7.** Lesion sensitivity and precision from the best models from the previous analysis, state-of-the-art models from SCT, and the results reported for an automated tool in a clinical evaluation study.

METHOD	Training Set Scale	Sensitivity	Precision
LesionSCynth (75%/25% Real/Synthetic)	17	$0.79^{a}$ ± 0.05	$0.53^{b}$ ± 0.06
LesionSCynth (50%/50% Real/Synthetic)	145	$0.81^{a}$ ± 0.04	$0.56^{b}$ ± 0.06
Real – Balanced (50%/50% Lesions/No Lesions)	145	$0.79^{a}$ ± 0.08	$0.56^{b}$ ± 0.07
LesionMix*_pop_* (50%/50% Real/Synthetic)	145	$0.83^{a}$ ± 0.02	$0.59^{b}$ ± 0.02
sct_deepseg_lesion -c t2 (v6.5)		0.41	$0.55^{b}$
sct_deepseg -task seg_ms_lesion (v6.5)		$0.79^{a}$	0.42
Lodé et al. (2025) (as reported in that paper)		$0.89^{a}$	$0.54^{b}$

To be able to compare with the results from Lodé et al. (2025), we used the metric definitions from that study, that is, a GT lesion is a true positive if there is a non-zero overlap with a prediction. For the results presented here, a standard threshold of 0.5 was applied to the softmax outputs of the models in the current study to match sct_deepseg -task seg_ms_lesion. Note, however, that Lodé et al. (2025) optimised their threshold to achieve a higher sensitivity. Results for the current study are presented as mean ± std. dev. over five runs. Statistical tests were conducted comparing the models from the current study with each other and with the external models. Only the overall metric values were reported in Lodé et al. (2025), that is, no image-level results are available, so we used the average metric values for all statistical tests here. To approximate the standard deviation for those results and also for the SCT results, we used the maximum standard deviation for the four models from the current study. Two-sample t-tests were then used for all pairwise comparisons. If two experiments share the same superscript letter in the table, this indicates that the difference between them is *not* statistically significant (p<0.05).

From the models in the current study, we see again that ${\text{LesionMix}}_{pop}$ trained at the highest training set scale achieved the best performance, with a mean lesion sensitivity of 0.83 and lesion precision of 0.59. We note that this is a lower sensitivity and higher precision than the model in the clinical evaluation study (sens = 0.89, prec = 0.54), but adjusting the threshold on the softmax outputs may allow us to get closer to these values. This demonstrates that, even though the metric values in the previous sections seem low, for example, compared a typical Dice score for brain lesion segmentation, models at this level of performance can still be impactful in a clinical setting, given the difficulty of the task.

From Table 7, we can also see that the newer lesion segmentation model from SCT (sct_deepseg seg_ms_lesion) achieved a much higher sensitivity than the previous version, with a sensitivity in a similar range to the models trained in this study. However, the precision was lower than the models from this study, which ranged from a precision of 0.53 to 0.59.

## 6 Discussion

### 6.1 Comparison with real-only baselines

As observed in Section 5.2, when few annotated volumes with lesions are available for training, introducing synthetic lesions with our method can significantly outperform training with real data only. A surprising and promising result was observed in Section 5.4 in the experiments with different ratios of synthetic to real examples at each epoch. Here, training with 25% synthetic lesion examples and 75% real examples per epoch at the lowest training set scale achieved an average FROC of 0.554, which is higher than all the other experiments with our method and higher than all baselines trained on real data only, regardless of the training set size. The standard error for this experiment is relatively large over the five runs, with the lowest FROC at 0.469 and the other four runs having a FROC between 0.558 and 0.603. This suggests that the particular volumes in the training set can have a large impact on the score when few volumes with real lesions are available. Nonetheless, this observation shows that, with a small training set and our method of lesion synthesis, it is possible to achieve the same, or possibly better, performance as having a much larger dataset for training.

A surprising result in Section 5.2 was that, when training on real samples only, adding volumes with no lesions to the training set led to a significant drop when training with 17 real volumes with lesions, but improved performance when the training set contained 145 real volumes with lesions. We speculate that at scale 17, the higher diversity of volumes without lesions relative to those with lesions led the model to learn a wider distribution for this group, which led to a reduced sensitivity at inference. Finally, *Real – Lesions Only* was not superior in all aspects to *Real – Balanced* at scale 17, as we saw in Table 2 that the latter achieved a higher lesion precision and thus higher F1 score.

In Section 5.2, we observed two cases where performance did not increase when we increased the training set size, including a drop from scale 17 to 36 when training with LesionSCynth. Given that the standard error bars overlap significantly across the results for the three training set scales, it is possible that this pattern is due to chance, and that if the same experiments were repeated many times with different random seeds, we might see a more intuitive pattern of increasing performance as we increase the training set size. We do observe the same pattern of higher performance at scale 17 versus 36 in Figure 11, with different proportions of real versus synthetic lesion MR volumes, but again the range indicated by the standard error is relatively high there. Another explanation of the pattern might be that the particular samples in the training set at scale 17 are “better” in some way than the set at scale 36, for example, more similar lesion characteristics to those in the held-out test fold, or there is some label noise introduced at scale 36 in the form of missing lesions in the GT mask. However, the same volumes with lesions are used for all 3 methods in Figure 7, and for the other 2 cases, performance does increase from scale 17 to 36. Therefore, it is more likely here that the combination of synthetic lesions with the particular real lesions at scale 17 achieves a better generalisation to the lesions in the held-out test set. However, given the large observed standard errors, it is not possible to make a concrete conclusion on these observations.

### 6.2 Comparison with other lesion synthesis methods

The results in Section 5.3 demonstrated that our method generally outperforms state-of-the-art lesion synthesis methods which are based on copying real lesions from one volume to another. The improvement of our method over the baselines decreases as we scale up the training dataset, and at the highest training set scale, a previous synthesis method, ${\text{LesionMix}}_{pop}$ , achieves the highest performance. CarveMix, however, remains consistently lower than both methods across all training set scales. At higher training set scales, therefore, it would seem that ${\text{LesionMix}}_{pop}$ is a better choice. Moreover, as the training set size increases, GANs and diffusion models become a more viable option for synthesis.

Why does LesionSCynth outperform ${\text{LesionMix}}_{pop}$ when few real data are available but the opposite is true with a large training set? The answer to this is unclear, but we can speculate as to the potential causes. We offer two hypotheses for why LesionSCynth outperforms ${\text{LesionMix}}_{pop}$ with a small training set: first, it may be that with a small number of real lesions, the segmentation model could overfit to the copied lesions from LesionMix, whereas LesionSCynth could create a wider range of intensity profiles. Second, both LesionMix and CarveMix introduce some hypointense synthetic lesions, even after standardising the source and target image intensity distributions. The impact of label noise has been shown to decrease with training data size (Rolnick et al., 2018), so these poor-quality lesions may have a larger effect on training when paired with a small set of real data compared with when many real examples are available. Then, for a much larger training set, the potential impact of overfitting or noise could be lessened. At the same time, LesionMix can create more realistic lesions in some aspects, especially texture, since they are based directly on real lesions. Moreover, LesionMix could create more diverse synthetic lesions when a large number of real lesions are available, and also because more extensive augmentation operations are applied by LesionMix than we applied to the candidate lesion masks with LesionSCynth.

One advantage of a parametric method such as ours over methods which copy existing lesions into other volumes is that the parameters could be adapted for domain adaptation or generalisation to other MR sequences. This could be done even without training on real lesion examples in the other MR sequences by instead drawing on prior knowledge. For example, previous studies have shown that lesions in STIR acquisitions tend to have higher contrast (Philpott & Brotchie, 2011), so we could increase the mean of the contrast distribution (μ=0.17 from Section 3.1), using a small validation set with STIR lesions to determine a reasonable value. For T1-w acquisitions where lesions appear hypointense, we could similarly adjust the contrast calculation so that μ=0.17 would mean that synthetic lesions have 17% *lower* intensity than their neighbourhoods, on average.

We note that there are several parts of the original LesionMix that are not applied in this study, notably the other branch which also randomly removes existing lesions through inpainting as an extra augmentation technique and also the use of a prior probability map to choose new lesion positions. These elements are excluded here as we chose to isolate the impact of synthesis of lesion appearance, and those techniques are agnostic of the synthesis method used and so could also be added to our method to potentially improve performance.

### 6.3 Method component analysis

Although our three-part approach to synthesising lesions allows us to create more realistic-looking lesions, one can ask whether synthetic lesions need to be completely realistic to benefit the trained segmentation model. Indeed, our ablation study presented in Section 5.4 indicates that simply increasing the intensity by a multiplicative factor to create a lesion may be enough to guide the model to learn a wider representation of lesion characteristics. Inducing a spatial gradient in the intensity and increasing local homogeneity of intensity did not further increase performance when training at the lowest training set scale.

One of the proposed advantages of the method is from the introduced diversity, both in the characteristics of the lesions given that the parameters for each lesion are drawn from random distributions, and in the tissue surrounding lesions, given that lesions are inserted in random positions into a random selection of volumes originally containing no lesions. However, as we observed in Section 5.4, there is a limit to the benefit from diversity. Indeed, inserting a fixed set of synthetic lesions into a fixed subset of volumes achieves similar performance to randomly sampling among 70 volumes with no lesions at each epoch, and synthesising new lesions on-the-fly. However, we can imagine situations where more no-lesion volumes might still be beneficial, if these contain types of acquisitions otherwise under-represented, for example, different scanners, resolutions, or even different MRI sequences.

Initial analyses showed that synthetic lesions increased lesion coverage in under-represented areas (see Supplementary Material), and we, therefore, conducted further experiments to determine whether this was the primary mechanism for the improvement in performance from LesionSCynth. The results of these experiments suggest that varying the lesion position was necessary but not sufficient to achieve the best performance. Nonetheless, previous work has shown that the synthetic data help to improve performance for under-represented modes in the original dataset—for example, Debs et al. (2020) found that simulation of patient-specific arterial input functions improved detection of infarctions through better coverage of rare physiological cases. In a similar way, our synthesis pipeline improves spatial coverage and could also be adapted to specifically target under-represented lesion characteristics where initial models perform poorly (e.g., lesions close to CSF).

### 6.4 Outlook

In Section 5.5, we observed that LesionSCynth, trained on 17 volumes with real lesions and 70 volumes with no lesions, outperformed a state-of-the-art model from the SCT. This difference in performance is likely due in large part to the similar distribution between our training and testing data. The acquisitions in the test set from Lodé et al. (2025) were extracted from OFSEP, as were a large part of the acquisitions in the training set for the current study (note, however, that there is no overlap between the subjects from these two sets). Therefore, both sets contain typical sagittal T2-w acquired in clinical practice in France, as well as similar distributions of resolution and even acquisitions from the same scanners. Moreover, both sets of manual annotations were reviewed and validated by the same senior neurologist (A.K.), so there may be more annotation shift between the SCT training set and the clinical evaluation study than for our training set. However, it is very promising that, given an in-distribution dataset with fewer than 20 volumes with real lesions, plus a larger set of volumes with no lesions, it is possible to achieve similar or better performance than training on a large out-of-distribution dataset containing over 1000 T2-w acquisitions.

Although the metric values may seem low when compared with brain lesion segmentation, spinal cord lesion segmentation is a more difficult task and suffers higher inter-rater variability (Lodé et al., 2025; Walsh et al., 2023). The best models from the current study achieved similar results to the model used by Lodé et al. (2025), albeit with a lower sensitivity and higher precision. Lodé et al. (2025) found that the outputs of the model in that study helped to improve the mean sensitivity of 20 radiologists and neurologists by 5 percentage points, and also led to an improvement in mean precision among raters with less than 10 years’ experience. Given that the models in the current study achieved similar performance, they may also be beneficial in a clinical setting.

Finally, LesionSCynth could also be used as a domain adaptation or generalisation technique. Given a set of manually annotated acquisitions from one scanner or one MR sequence and a set of acquisitions from another scanner or sequence of healthy controls or people with MS who do not have spinal cord lesions, the LesionSCynth parameters could be adapted to synthesise lesions in the new domain, which could increase the applicability of the trained segmentation model. Moreover, such a technique could also be used in an active learning scenario, where a small set of manual annotations are used to train an initial model, whose outputs are then given to experts to manually correct.

### 6.5 Limitations and future work

We believe that LesionSCynth is a good first step in spinal cord lesion synthesis, as it is motivated by key observations of real lesions, and we demonstrated that it can significantly improve lesion detection when few annotated examples are available for training. However, the method makes several simplifying assumptions that could be refined. For example, LesionSCynth assumes a smooth isotropic Gaussian intensity profile, which does not capture the irregular, multi-lobed, or elongated morphologies frequently observed in clinical data. Models trained on overly smooth lesions may fail to generalise to these more complex cases. Moreover, modelling the intensity profile isotropically ignores important potential differences in intensity gradients between sagittal and axial acquisitions. Future work could address these points by (i) generating more diverse and complex lesion shapes, such as lesions with multiple lobes and multiple modes, and long lesions with high intensity along the centreline of the long axis; (ii) modelling different lesion textures; and (iii) investigating whether distinct approaches should be taken for sagittal versus axial acquisitions. For the latter question, a simple first step could be to examine retaining anisotropic spacing in the preprocessed images and synthesising single-slice lesions. Another potential approach to including more diverse intensity profiles would be to model intensity versus distance to the lesion boundary rather than to its centre of mass. For each of these questions, though, in-depth analysis is required to assess the frequency and distribution of each type of pattern in real lesions.

The method is based on empirical observations on (1) the contrast of lesions compared with neighbouring normal-appearing tissue, (2) the spatial variation of intensity within lesions, and (3) the variance of intensities within lesions. We apply independent operations to emulate these observations and, therefore, the method implicitly assumes independence of these aspects, which is not necessarily the case. It is likely, for example, that lesions with a higher level of spatial variation also have a higher overall intensity variance, and it is possible that lesions with higher contrast have higher variance. Further research would be required to analyse these relationships and adapt the method, if required. However, ablation experiments indicated that extra steps inducing spatial variation or reducing overall variance do not necessarily bring more benefit than simply increasing the intensity within a lesion candidate by a multiplicative factor, even if they do help to make more realistic lesions, so it is not clear that future work in this direction would bring further benefit.

The current study takes a deliberately simple approach to lesion shape and position, using augmented existing real shapes from the training dataset and randomising the lesion position. Further work on modelling shapes and using probabilistic heatmaps of lesion occurrence derived from real datasets could be used to guide placement along the cord (e.g., cervical vs. thoracic regions), or lesions could be placed relative to local anatomical landmarks. Moreover, LesionSCynth currently relies on preprocessing the data to shift around the spinal cord centreline so that all volumes are roughly aligned. While this facilitates model training, it may contribute to the lower performance observed when training only on synthetic lesions. Extending the method with a more sophisticated placement approach in the original volumes would, therefore, be an interesting direction to potentially further improve performance.

We have claimed that the improved performance with synthetic lesions is likely due to greater diversity in lesion characteristics (intensity, location, and shape), but we have not demonstrated explicitly that this is the cause. Other potential causes include a reduction in label noise, given that we are explicitly inserting hyperintensity inside lesion masks, or differences in lesion prevalence. We have attempted to rule out this latter reason as a cause by assessing the effect of different ratios of lesion to no-lesion volumes when training on real data only. All in all, concluding on the exact cause(s) for improvements when using synthetic lesions remains an open question. Future work could investigate this more directly, for example, by quantitatively comparing the distributions of lesion shape, intensity, and spatial location in real versus synthetic lesions.

Finally, the approach we took of inserting synthetic lesions into volumes without lesions assumes that these volumes are available, even in scenarios with few data annotations. We believe this a fair assumption to make, given that there are already open-source spinal cord datasets with acquisitions of healthy subjects (Cohen-Adad et al., 2021), and in clinical databases, radiologist reports associated with the acquisitions could be used to identify acquisitions containing no lesions.

### 6.6 Key takeaway points and recommendations

- Given a training dataset with few real lesions (17 volumes with real lesions), training a deep learning segmentation model with real and synthetic lesions achieved similar performance to a model trained only on real data including 145 volumes with real lesions. The best result was achieved when each epoch contained 75% real lesions and 25% LesionSCynth synthetic lesions, so we recommend researchers to use this approach if only a small annotated training set is available.
- Given larger training datasets (145 volumes with real lesions), LesionSCynth did not improve a segmentation model over using real data alone, but also did not deteriorate performance. At this training set size, ${\text{LesionMix}}_{pop}$ achieved the best result and so may be a more appropriate choice for lesion synthesis with large training sets.
- We observed no difference in performance when using our one-step approach of increasing contrast with a multiplicative factor and our three-step approach which creates more realistic lesions, so for simplicity, researchers can use the one-step approach. We note though that the difference in computation time between the two approaches is negligible compared with the overall time for training a deep learning model. Future work could investigate expanding our full method to more complex patterns and more sophisticated lesion placement strategies to seek further improvements.
- Generating diverse synthetic examples on-the-fly during training led to no improvement over using a smaller set of fixed examples matching the number of volumes with real lesions. For simplicity, researchers can use a small set of synthetic lesions with our method. The potential advantage of large sets of synthetic lesions should be investigated, however, if seeking to increase representation of under-represented characteristics using non-lesion acquisitions from those groups (different sites, populations, etc.).

## 7 Conclusion

This study introduced LesionSCynth, a parametric lesion synthesis method that improves MS lesion segmentation in spinal cord MRI, particularly when annotated data are limited. Our experiments showed that models trained with synthetic examples and a small number of real lesions can match or exceed the performance of models trained on much larger real-only datasets. LesionSCynth outperforms existing synthesis methods at smaller data scales and offers flexibility for domain adaptation through adjustable parameters. Despite its simplicity, the method provides substantial gains in model performance, making it a practical tool for enhancing spinal cord lesion detection and reducing annotation effort.

## Supplementary Material

Supplementary Material

## Data Availability

Most of the data used in this study are from the French MS Registry (OFSEP), and requests can be made directly to OFSEP in the form of a project proposal.^2^ Code for the project is available on GitHub.^3^

## Footnotes

1
https://portal.fli-iam.irisa.fr/ms-multi-spine/
