# Supplementary material for: LesionSCynth: A simple parametric lesion synthesis method to improve spinal cord lesion segmentation in low-data scenarios
Source: Imaging Neurosci (Camb). 2025 Nov 26;3:IMAG.a.1029. doi: 10.1162/IMAG.a.1029 (PMC12658772; doi:10.1162/IMAG.a.1029)
Supplement: Supplementary Material [file IMAG.a.1029_supp.pdf]

A Supplementary Material

A.1 Preprocessing

A.1.1 Spinal Cord Segmentation

A key part of preprocessing is to obtain a segmentation mask of the spinal cord. This is later used in 1) cropping the image to remove redundant information, 2) calculating the contrast between a lesion and its neighbouring spinal cord voxels, and 3) ensuring inserted synthetic lesions are only within the spinal cord. Spinal cord segmentations were obtained using the trained deep learning models from the Spinal Cord Toolbox (SCT) (De Leener et al., 2017).

The T2-w model[1] was used for both the upper and lower volumes. However, this model often failed to segment the conus medullaris (lower end) well and frequently descended below the terminal end of the spinal cord or segmented parts of the CSF. The available SCT lumbar T2-w model[2], on the other hand, performed well in the conus region but often did not capture the spinal cord at the top of the lower acquisitions. Therefore, we merged both segmentations for lower volumes to capture the best of both models. Specifically, we computed the midpoint of the lumbar model's segmentation along the superior-inferior axis and constructed the final mask by taking the lumbar model's segmentation below this point and the T2-w model's segmentation above it. The more recent update of the contrast-agnostic model from SCT (v6.4 and above) seems to perform well on both our upper and lower acquisitions from preliminary investigations, and so this model could be used in future to segment the spinal cord, replacing the current two-step approach.

We applied several postprocessing steps to the spinal cord masks to reduce the possibility of false positive segmentations outside of the spinal cord. First, we applied a morphological closing operation using a cube-shaped structuring element with a width of 3 voxels. This helped close small gaps in the segmentation which can occur occasionally, particularly along the S-I axis. Next, we removed all but the largest 3D connected component in the segmentation, and we applied a 2D binary opening to each sagittal slice, with a square structuring element of width 3 voxels. This helped to remove isolated parts of the segmentation which could cause unusual results when synthesising lesions. Finally, we observed several upper acquisitions where the spinal cord segmentation did not capture a small part of the bottom of the volume (e.g., 2-3 vertebrae). To address this, we extrapolated the segmentation into this region, given that the spinal cord shape tended to be approximately linear in this small section.

[1] `sct_deepseg_sc -c t2` (SCT v6.3) (Gros et al., 2019)

[2] `sct_deepseg -task seg_lumbar_sc_t2w` (SCT v6.3)

A.1.2 Orientation and Spacing

We converted all images and segmentation masks to LAS+ orientation and re-sampled to 0.5mm isotropic spacing. We used trilinear interpolation when re-sampling anatomical volumes and nearest neighbour interpolation for segmentation masks. Although the default setting of nnU-Net for anistropic data is to retain anistropic spacing, we found in previous experiments that isotropic spacing can lead to better results, which is also observed in a recent nnU-Net update (Isensee et al., 2024). Finally, the choice of 0.5mm is slightly lower than our median in-plane spacing of 0.58mm, but retains consistency with previous works on spinal cord lesion segmentation (Gros et al., 2019; Walsh et al., 2023, 2024).

A.1.3 Spinal Cord Centreline

We computed the spinal cord centreline by taking the centre of mass of the spinal cord segmentation in each axial slice. Where there were no voxels segmented in a given slice, we interpolated linearly from the nearest adjacent slices with segmented voxels. We extrapolated the centreline above and below by 10 slices to ensure that we captured the full cord as well as a small buffer zone. Finally we applied smoothing to the centreline, taking the vectors of x and y coordinates in each axial slice and applying a 1-D uniform filter with a window size of nine slices.

A.1.4 Crop and Shift

Finally, we cropped and shifted the image around the spinal cord centreline, an approach proposed by (Gros et al., 2019) and recently used by (Walsh et al., 2024). Concretely, for each axial slice, we cropped to a square patch of 48×48 voxels around the centreline point. The 2D slices were then stacked along the z-axis, leading to a 'straightened' spinal cord without introducing interpolation. Although shifting the image around the centreline does not necessarily lead to improved segmentation performance, doing so allowed us to reduce the training time by a factor of three compared to simply cropping around the bounding box of the whole spinal cord. Moreover, it simplifies the positioning and orientation of synthetic lesions as all volumes are approximately aligned.

A.2 Lesion Contrast & Intensity Variation

Contrast vs. Neighbourhood We chose to compare the lesion intensity to the intensity in its neighbourhood, rather than comparing to background or an average over the spinal cord, because a hyperin-

tense lesion is only detectable in the image if it appears as hyperintense with respect to surrounding local tissue, not relative to the image as a whole. Furthermore, the intensities of different tissues can vary over the image. For example, to unambiguously detect a small lesion in the region of visible grey matter in the spinal cord, the intensity must be higher than for a lesion in a more homogeneous, lower intensity region of the spinal cord. Moreover, field inhomogeneities can introduce bias and affect the calculation of average background or spinal cord intensity.

Effect of Neighbourhood Size Fig. 1 compares the different contrast distributions obtained when changing the area taken around each lesion, i.e., the radius of the structuring element used when dilating each slice. Although the contrast increases slightly on average as we increase the area around the lesion, the distributions remain largely similar. For the purposes of this study, we used a 5-voxel radius for the dilation structuring element.

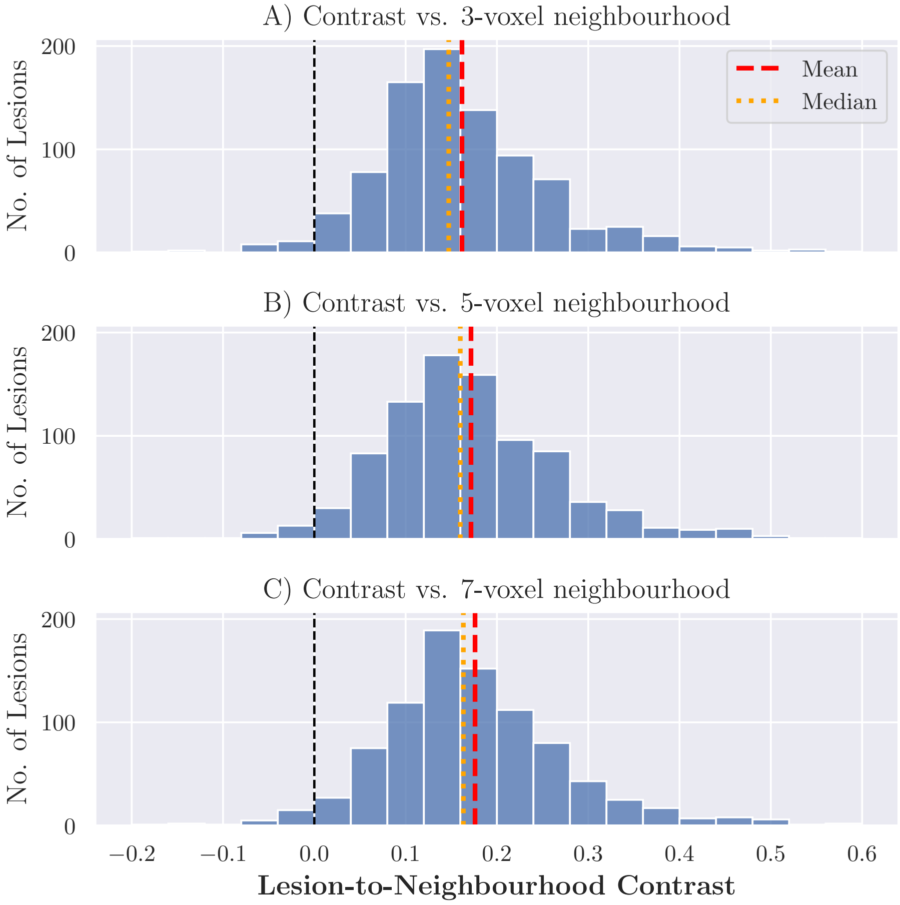

Figure 1: Distributions of lesions vs. their neighbourhoods, for different sizes of the dilation element used to obtain the neighbourhood masks, i.e., a disk structuring element with radius of A) 3 voxels, B) 5 voxels, or C) 7 voxels.

Variability of the Synthesis Parameters The most important parameters used to initialise the lesion synthesis method are the mean ($\bar{\mu}$) and standard deviation ($\bar{\sigma}$) of the contrast ratio among the real lesions in the training set – these were computed from the training set for a given experiment. Table 1 shows the variability of these computed contrast ratio statistics across the four different training set scales and the five runs at each scale. As might be expected, the variability of these parameters across the five runs increases for smaller datasets. However, even at low data set sizes, the difference between the minimum and maximum values for $\bar{\mu}$ (0.044) remains small relative to the spread of the distribution used for sampling a contrast ratio for synthesis (i.e., $\bar{\sigma} \approx 0.11$).

Table 1: Summary of the contrast ratio statistics computed on real lesions in the training set of a given experiment. The mean and standard deviation of the contrast ratio were used to initialise the lesion synthesis function.

	Mean Contrast Ratio ($\bar{\mu}$)		Std. Dev. of Contrast Ratio ($\bar{\sigma}$)	
Training Set Scale	Range (Min – Max)	Mean	Range (Min – Max)	Mean
17	0.142 – 0.186	0.161	0.103 – 0.113	0.108
36	0.157 – 0.183	0.169	0.099 – 0.111	0.105
72	0.165 – 0.174	0.168	0.096 – 0.105	0.099
145	0.168 – 0.175	0.171	0.092 – 0.103	0.097

Contrast Ratio Distribution in STIR To assess whether the contrast ratio of lesions to their neighbourhood follows a similar distribution in sagittal STIR volumes than in sagittal T2-w, we assessed 29 STIR volumes with manual segmentations created directly on the STIR volumes. These corresponded to a subset of subjects in the training dataset in this study. Fig. 2 shows the distribution of contrast ratio calculated for the manually segmented lesions in the STIR volumes. The mean across all of the lesions is higher than for sagittal T2-w, which aligns with findings in previous work that STIR can show higher lesion contrast than T2-w (Philpott & Brotchie, 2011). We note that the shape of the distribution is skewed right, although this might approach a normal distribution if the number of subjects and lesions were increased. The apply LesionSCynth in this context, the contrast ratio distribution could be replaced with a skew-normal distribution fitted to the real lesions in the training set, and the parameters of that distribution can be estimated with SciPy (`scipy.stats.skewnorm.fit()`).

Low Contrast Lesions As we can see from Fig. 1, there are several lesions with negative contrast or low contrast compared to their neighbourhoods. Low contrast lesions typically occur when: 1) the neighbourhood takes into account voxels with CSF partial volume effects (or normal tissue with high intensity, e.g., grey matter), 2) the lesion (or part of the lesion) is not clearly discernible in the sagittal T2-

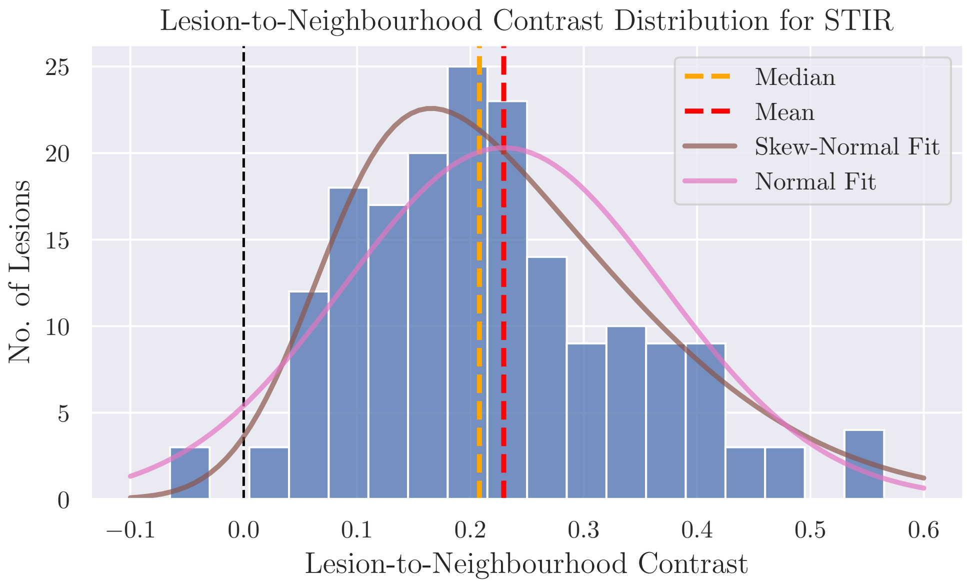

Figure 2: Distribution of contrast ratio for real lesions in 29 sagittal STIR volumes. A skew-normal distribution was fitted to the data and is overlaid here, parametrised as: $SN(\xi = 0.069, \omega = 0.214, \alpha = 3.338)$. A fitted normal distribution is also overlaid: $N(0.229, 0.141)$.

w acquisition but is evident in other acquisitions for the same subject, such as STIR or axial acquisitions, or 3) the manual segmentation misses some hyperintensity or includes some lower intensity voxels. Fig. 3 presents several examples of these.

Spatial Variation of Intensity The following is a more detailed explanation of the background analysis and processing used to arrive at the figure in the main paper which plots the average intensity vs. the distance from the lesion centroid, and shows that lesion intensity tends to be higher at the centre of the lesion.

When summarising intensity of lesion voxels across the whole dataset, especially when looking at it through the lens of spatial variation, it is important to consider the contribution of the normal tissue in the lesion region to intensity. In particular, if a lesion occurs in the region of CSF at the outer edges of the spinal cord, then partial volume effects may cause the lesion intensity to be higher in that region. Indeed, if we take the voxels of all lesions in the dataset and average them in the axial plane, we get the pattern shown in Fig. 4. The main observable pattern here is a ring of higher intensity around a darker centre, and the size and intensity pattern are comparable to the ring of CSF around the spinal cord for normal-appearing tissue. This is true in particular along the R-L axis, where resolution is limited, and so partial volume effects are more likely between CSF and lesions.

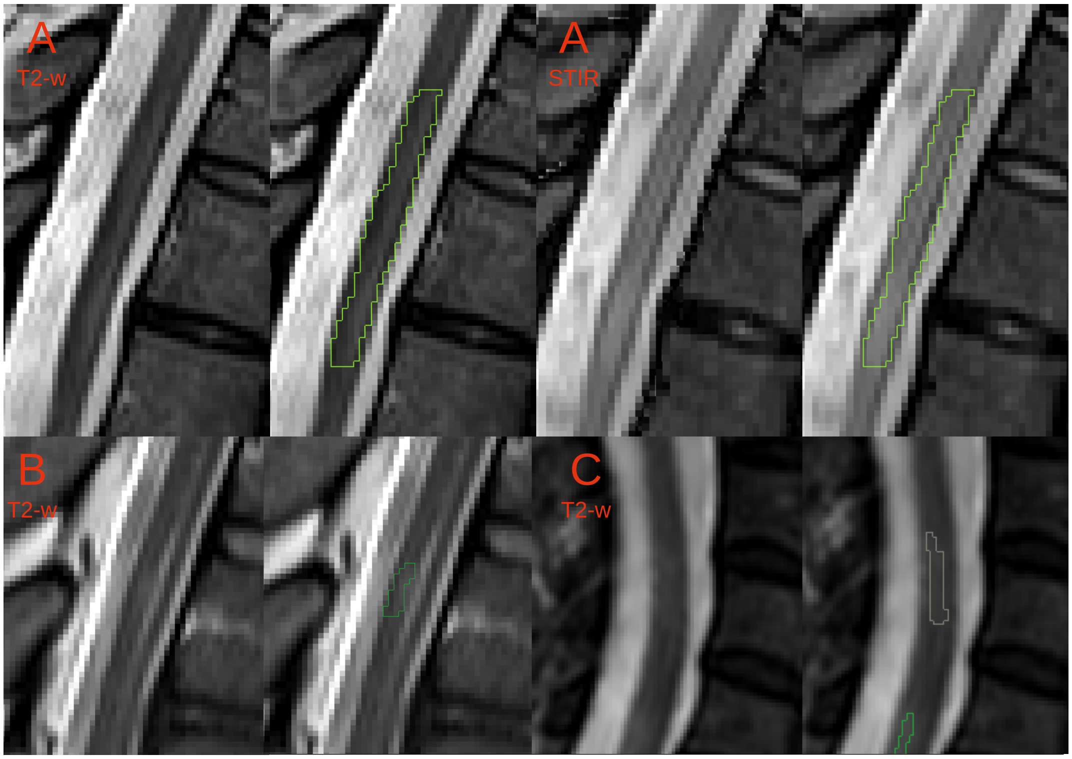

Figure 3: Examples of lesions in the ground truth annotations with low average contrast with surrounding tissue. Note that the intensity histograms have been clipped for the figure, to have better improve the visibility of the lesions. A) Not all of the segmented area contains hyperintensity in the T2-w image (average contrast = -0.06), though the hyperintensity is discernible in the STIR acquisition. B) The lesion occurs next to hyperintensity likely caused by the CSF-filled ependymal canal (average contrast = -0.005). C) The lesion occurs in the region of other hyperintensity, possibly grey matter or artefacts (average contrast = 0.002).

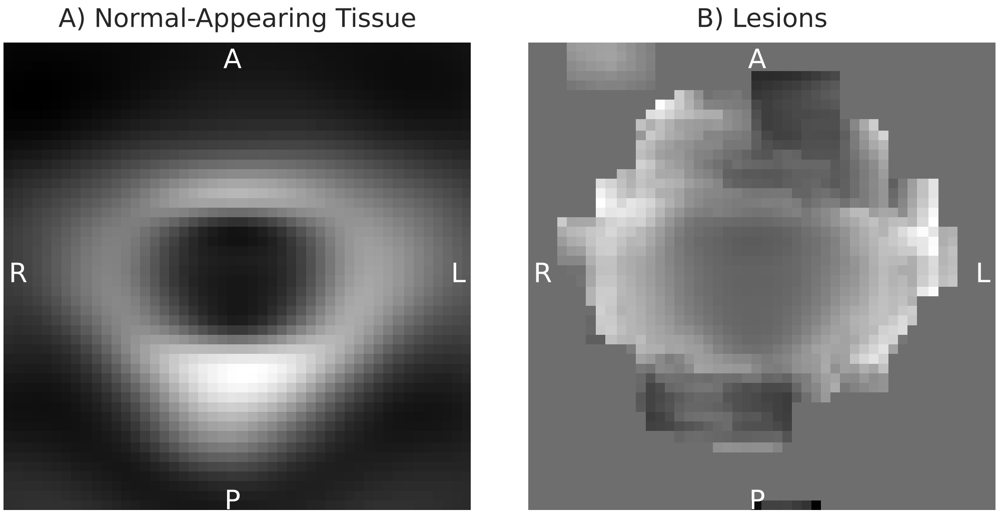

Figure 4: Average intensity of voxels overlaid on the axial plane. This was done after preprocessing, i.e., all volumes were shifted around the spinal cord centreline so that they align roughly in the axial plane. A) ten random patches of $48 \times 48 \times 48$ voxels containing normal-appearing tissue were extracted from all volumes in the dataset, the patches were each standardised to $\mu = 0, \sigma = 1$ and the average was taken along the z-axis. B) all lesions were extracted from the dataset in patches of $48 \times 48 \times b$ where b is the length of the lesion in the z-axis, the patches were each standardised to $\mu = 0, \sigma = 1$ and the average was taken along the z-axis, excluding any non-lesion voxels. The non-zero voxels at the edge of the image here were caused by an imprecise centreline during preprocessing, but only a small number lesions were affected.

Analysis of the relationship between intensity and relative position within a lesion may be dominated by this higher outer intensity caused by the CSF, and indeed we observed such an effect in an initial analysis of intensity and spatial variation. To eliminate this 'bias' from the analysis, we computed the average normalised intensity in the axial plane across many non-lesion patches, and then subtracted this from the corresponding voxels in the normalised lesions. We outline a justification below for this procedure to analyse the spatial variation of lesion intensity.

Given a small patch, P, containing normal-appearing tissue, we assume that the image intensity at a given point $\boldsymbol{x} \in P$ is a function $g_P(\boldsymbol{x})$ with the intensity determined principally by the underlying tissue. $g_P(\boldsymbol{x})$ also depends on the particular patch (and the acquisition) but we assume that this function $g_P(\boldsymbol{x})$ is drawn from the same distribution for all patches.

We assume that the intensity in a patch containing a lesion is given by

$$g^*_P(\boldsymbol{x}) = g_P(\boldsymbol{x}) \times (1 + f_P(\boldsymbol{x})) \tag{1}$$

where $f_P(\boldsymbol{x})$ is zero everywhere outside of a lesion and inside the lesion determines the hyperintensity

profile, but we make no further assumption on f at this point. We aim to isolate the effect of this $f_P(\boldsymbol{x})$ on observed intensities, given that simply taking the raw average across lesion patches is dominated by the higher intensities of CSF (which would be captured by $g_P(\boldsymbol{x})$).

Standardising a 'normal' patch and a 'lesion' patch to mean 0 and standard deviation 1 gives the following:

$$z_P(\boldsymbol{x}) = \frac{g_P(\boldsymbol{x}) - \mathbb{E}_{\boldsymbol{x}\in P}(g_P(\boldsymbol{x}))}{s_{\boldsymbol{x}\in P}(g_P(\boldsymbol{x}))} \tag{2}$$

$$z_P^*(\boldsymbol{x}) = \frac{g_P(\boldsymbol{x}) - \mathbb{E}_{\boldsymbol{x}\in P}(g_P(\boldsymbol{x})) + g_P(\boldsymbol{x})f_P(\boldsymbol{x}) - \mathbb{E}_{\boldsymbol{x}\in P}(g_P(\boldsymbol{x})f_P(\boldsymbol{x}))}{s_{\boldsymbol{x}\in P}(g_P(\boldsymbol{x})f_P(\boldsymbol{x}))} \tag{3}$$

where s^2 denotes the sample variance.

We assume that $s_{\boldsymbol{x}\in P}(g_P(\boldsymbol{x})) \approx s_{\boldsymbol{x}\in P}(g_P(\boldsymbol{x})f_P(\boldsymbol{x}))$ given that 1) f is non-zero for only a subset of points, and 2) the high intensity of CSF and low intensity of normal-appearing tissue will dominate in the calculation of variance.

We can then take the mean of the z-scores for normal-appearing tissue over a large number of patches to estimate the expected 'normal' intensity in the axial plane (shown in Fig. 4A), denoted as $\mathbb{E}_P(z_P(\boldsymbol{x}))$, and subtracting this from the normalised lesion intensities yields

$$z_P^*(\boldsymbol{x}) - \mathbb{E}_P(z_P(\boldsymbol{x})) \approx z_P(\boldsymbol{x}) - \mathbb{E}_P(z_P(\boldsymbol{x})) + \frac{g_P(\boldsymbol{x})f_P(\boldsymbol{x}) - \mathbb{E}_{\boldsymbol{x}\in P}(g_P(\boldsymbol{x})f_P(\boldsymbol{x}))}{s_{\boldsymbol{x}\in P}(g_P(\boldsymbol{x})f_P(\boldsymbol{x}))} \tag{4}$$

Taking the expectation over many lesion patches, $\mathbb{E}_{\boldsymbol{x},P}[z_P(\boldsymbol{x}) - \mathbb{E}_P(z_P(\boldsymbol{x}))] = 0$, and so we are left with the normalised $g_P(\boldsymbol{x})f_P(\boldsymbol{x})$ term.

In the main paper, we plot $\mathbb{E}_P[z_P^*(\boldsymbol{x}) - \mathbb{E}_P(z_P(\boldsymbol{x}))]$ vs. the distance from the lesion centroid for all points in all lesions in the dataset. Originally, when using $\mathbb{E}_P(z_P^*(\boldsymbol{x}))$ as the intensity quantity on the y-axis, we observed that the intensity inside lesions tended to increase with increasing distance from the centroid, though this is likely because of the underlying tissue intensity variation.

If $f(\boldsymbol{x})$ were constant, i.e., $f(\boldsymbol{x}) = c, \forall \boldsymbol{x}$, then we would expect that using $\mathbb{E}_P[z_P^*(\boldsymbol{x}) - \mathbb{E}_P(z_P(\boldsymbol{x}))]$ as the quantity on the y-axis of this intensity-distance plot would yield a similar pattern, albeit scaled, to using $\mathbb{E}_P(z_P^*(\boldsymbol{x}))$, based on Eq. 4. Furthermore, if $f(\boldsymbol{x})$ tended to increase intensities further from the lesion centroid, then this would amplify the underlying effect of $g_P(\boldsymbol{x})$, and so we should again see a similar pattern to that originally observed, i.e., increasing intensity with increasing distance.

Therefore, given that plotting $\mathbb{E}_P[z_P^*(\boldsymbol{x}) - \mathbb{E}_P(z_P(\boldsymbol{x}))]$ in the figure in the main paper shows that the

transformed intensity is highest at the lesion centroid and then decreases, this suggests that this spatial pattern is largely driven by $f(\boldsymbol{x})$, i.e., that the intensity increase within lesions tends to be higher at the centroid and decreases towards the edges.

A.3 Synthetic Lesion Examples

Fig. 5 presents random examples of real lesions and synthetic lesions generated by LesionSCynth, LesionMix, and CarveMix. The 20 examples for each of the four categories were selected completely at random, and no manual curation was done. Note that these examples were generated and visualised after preprocessing, i.e., shifting the image around the spinal cord centreline, which can introduce some artefacts, e.g., the jagged edges of CSF that can be observed. Moreover, at the end of the spinal cord, the preprocessed images can sometimes appear slightly warped, given that the spinal cord tapers out in this region, and so the exact centreline can be noisier from one axial slice to the next, and the segmentations can be less precise in general.

Comparing the three synthesis methods, we see that our method generated examples closer in appearance to the real lesions. In particular, both LesionMix and CarveMix sometimes generated lesions which were hypointense relative to the image, even after normalising all images to mean 0 and standard deviation 1. Moreover, CarveMix sometimes included areas from the original images around the real lesions, which introduced further unrealistic elements. For example, in several examples, parts of CSF in the source image were included at the edge of the inserted area (e.g., last row, fourth from last column).

For cases where LesionSCynth lesions are not easily discernible in the figure, it may be because 1) a low contrast factor was sampled, or 2) the candidate lesion mask was mostly outside the spinal cord and only the small part inside the spinal cord at the CSF boundary was kept. However, most inserted lesions are clearly visible in the figure, and moreover, some of the real lesion examples are also not quite visible.

A.4 Lesion Position

One of the ways lesion synthesis could help train more robust models with small training sets is by expanding the spatial distribution of lesions. Fig. 6 seeks to examine differences in lesion distribution between sets of real lesions and synthetic lesions created by LesionSCynth. During synthesis, the position of lesions is randomised along the caudal-rostral direction (y-axis in Fig. 6). In this way, the synthesis process can increase variability in lesion placement and could lead to more robust segmentation in under-represented locations in the training dataset. For example, we can see that real lesions are more common

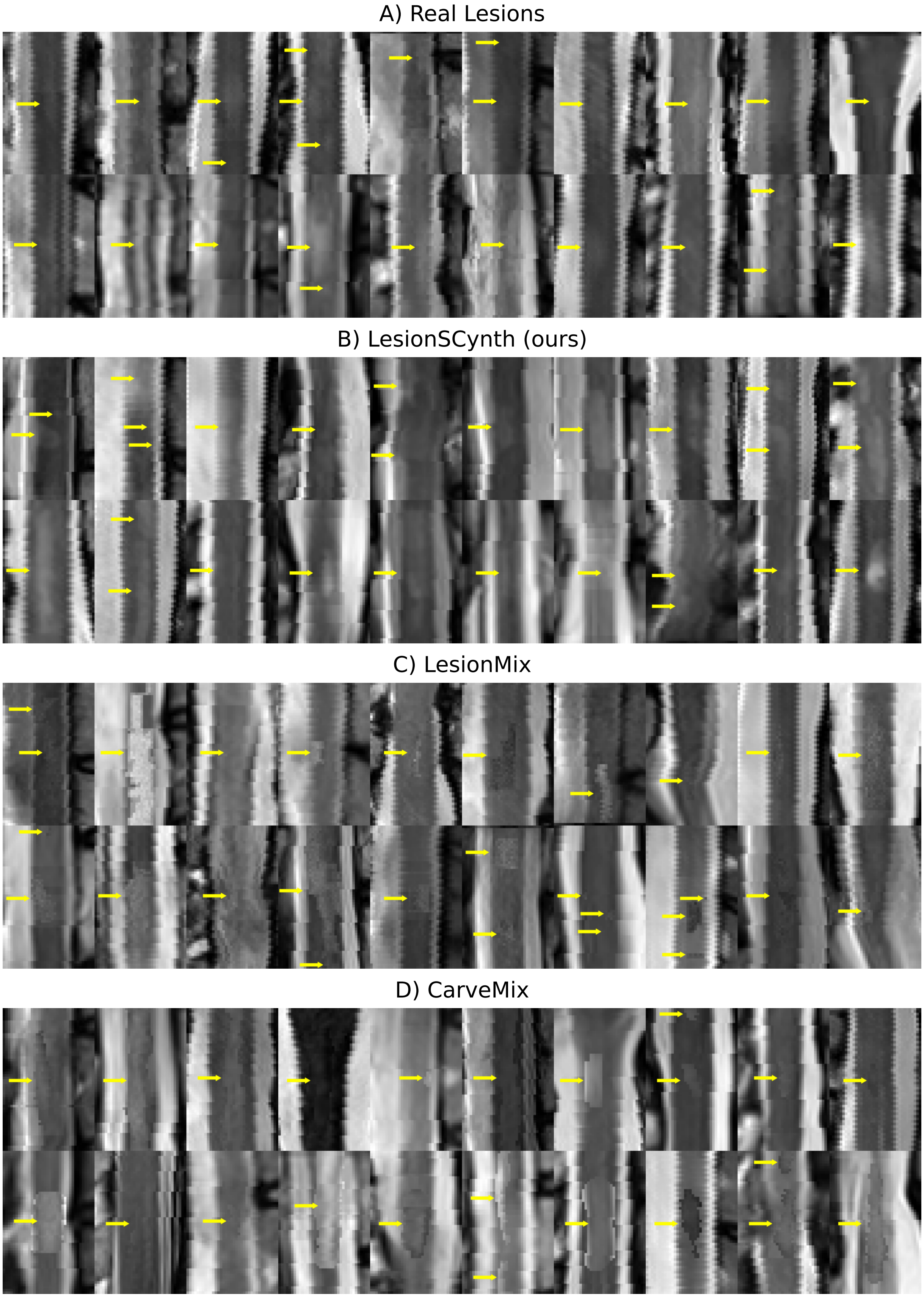

Figure 5: A random selection of real lesions and synthetic lesions from our method and the other two synthesis methods studied in this paper. See Sec. A.3 for more details and discussion.

in the cervical cord in Fig. 6 but the synthetic lesions for these particular generated sets tended to be more common in the upper thoracic cord. On the other hand, one could also argue that synthetic lesions should be more representative of reality, and that lesion placement should be constrained by priors using probabilistic heatmaps of real cases, which could enable the model to better learn typical lesion locations. In-depth analysis and tests are necessary to conclude on this point and would be an interesting direction for future work.

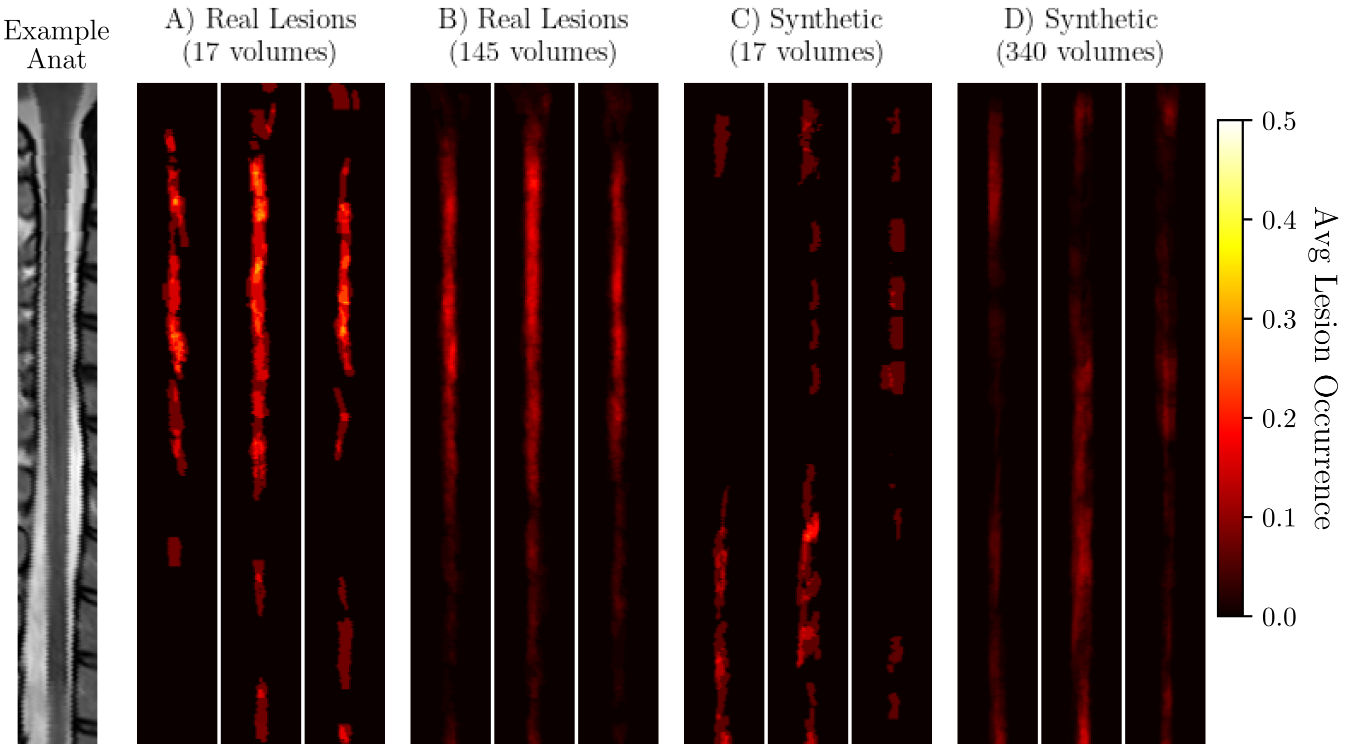

Figure 6: Heatmaps of lesion probability per voxel among volumes with lesions. The first image is an anatomical image provided simply for reference as to the corresponding vertebral level etc. The heatmaps are shown for three sagittal slices of the preprocessed volumes: the 18th, 24th, and 30th slice (out of 48 slices). The analysis is limited to the upper section of the cord to ensure consistent coverage across all volumes.
A) Heatmap computed from 17 volumes with real lesions, corresponding to one of the five folds used for training at the lowest training set scale. **B)** As before, but corresponding to one of the folds when training at the highest training set scale. We can see that the distribution is smoother than for only 17 volumes, but the higher probability of cervical lesions is still present. **C)** Heatmap for random synthetic lesions inserted into 17 random volumes with no lesions. There is a higher representation of lesions in the lower part of the image. **D)** Heatmap for multiple versions of random synthetic lesions inserted into 100 volumes with no lesions. A random sample of 340 examples was taken to represent a potential 20 epochs of synthetic examples when training at the lowest training set scale (17).

A.5 Additional Results

Classifying Real vs. Synthetic Lesions Training a segmentation model on data with only synthetic lesions led to poor test performance (FROC=0.22) compared to training on a small set of real lesions (FROC=0.44), indicating that the distributions of these two sets are still distinct. We further assessed this by training a classifier to to distinguish between normal tissue, real lesions and synthetic lesions. The classifier was based on the same architecture as the encoder of the U-Net segmentation model, with global average pooling and two fully connected layers (320 nodes for the first layer and 3 nodes for the output layer) added at the end of this encoder. The classifier was trained on small patches of $48 \times 48 \times 48$ voxels from 236 volumes, either of normal-appearing tissue or centred on a real or synthetic lesion. The model was trained for 500 epochs with a batch size of 64, where an epoch contained 6 patches from each subject (50% normal tissue, 50% real or synthetic lesion), and synthetic lesions were inserted only into volumes without real lesions (as when training a segmentation model in the main paper). Other hyperparameters were the same as the segmentation model used in the main paper.

The classifier was tested on lesion or normal-appearing tissue patches from 44 volumes, and resulted in the confusion matrix shown in Table 2. Many of the synthetic lesions were distinguishable from real lesions by the classifier – 17 of the 23 synthetic lesions were correctly classified as synthetic, whereas only 6 were misclassified as real lesions. This indicates that the distributions of these two sets remain distinct, and further work on reducing this difference may improve the performance of a segmentation model trained with both real and synthetic lesions. In particular, improving this aspect would likely improve upon the poor performance observed when training a segmentation model using *only* synthetic lesions.

Table 2: Confusion matrix of classifier predictions.

		**Predicted Class**			
		Normal	Real Lesion	Synthetic	Sensitivity
**Ground-Truth Class**	Normal Tissue	35	13	4	$^{35}/_{52} = 0.67$
	Real Lesion	9	92	0	$^{92}/_{101} = 0.91$
	Synthetic Lesion	0	6	17	$^{17}/_{23} = 0.74$
	Precision	$^{35}/_{44} = 0.80$	$^{92}/_{111} = 0.83$	$^{17}/_{21} = 0.81$	

Optimiser settings We report the results on the full test set for two different optimiser settings at the lowest training set scale (17) in Table 3. For these experiments, using the AdamW optimiser with no learning rate scheduler and GroupNorm as the normalisation used in the network outperforms or equals the default setting of nnU-Net of SGD optimiser with polynomial learning rate scheduler and InstanceNorm. As mentioned in the main paper, since the AdamW/GroupNorm setting performed better

Table 3: Comparison between AdamW setting and nnU-Net SGD at training set scale 17. Mean and standard deviation are taken over five runs.
Statistically significant difference between SGD and AdamW setting at: *0.05 significance, **0.01 significance.

Method	Setting	$FROC_{max}$	
Real – Balanced	SGD + InstanceNorm	**0.372**	± 0.024
	AdamW + GroupNorm	0.365	± 0.052
Real – Lesions Only	SGD + InstanceNorm	0.368	± 0.067
	AdamW + GroupNorm	**0.455***	± 0.061
LesionSCynth (Ours)	SGD + InstanceNorm	0.425	± 0.013
	AdamW + GroupNorm	**0.523****	± 0.036

than SGD/InstanceNorm on the validation folds across all three experiments, we used this configuration for all other experiments reported in the paper.

Evaluation at thresh=0.5 Finally, Table 4 complements the results presented in the paper with further evaluations at a threshold of 0.5 on the softmax scores. One might have expected that training only on volumes containing lesions vs. balancing volumes with lesions with volumes with no lesions would have some effect on the balance between sensitivity and precision, as observed previously in studies on class imbalance (e.g., Walsh and Tardy (2023). However, a large effect is not observed when comparing *Real - Lesions Only* and *Real - Balanced*. Moreover, we observe that LesionSCynth generally leads to higher lesion sensitivity than the real-only baselines at thresh=0.5, but the precision is lower than for the baselines at higher training set scales (although these differences in precision are mostly not statistically significant).

Table 4: Lesion sensitivity and lesion precision at a binarisation threshold of 0.5 on the softmax scores, to complement the results in the main text where the models were evaluated at a threshold which yields FPPI=1. **Real - Lesions Only**: Baseline trained on only real volumes with lesions; **Real - Balanced**: Baseline trained on real volumes, balanced per epoch between volumes with real lesions and volumes with no lesions; **LesionSCynth**: trained on a mix of volumes with real lesions and synthetic lesions (balanced per epoch).
**: 0.01; *: 0.05 – statistically significant difference, comparing to LesionSCynth at the same scale.

Training Set Scale	Method	Lesion-wise Sensitivity	Lesion-wise Precision
17	Real - Lesions Only	.55 ± .05**	.24 ± .07**
	Real - Balanced	.49 ± .08**	.28 ± .07**
	LesionSCynth (Ours)	**.66** ± .02	**.29** ± .04
36	Real - Lesions Only	.63 ± .04**	.27 ± .03**
	Real - Balanced	.60 ± .06**	.30 ± .05**
	LesionSCynth (Ours)	**.66** ± .02	**.34** ± .02
72	Real - Lesions Only	.66 ± .04*	.34 ± .04**
	Real - Balanced	.64 ± .04**	**.35** ± .06
	LesionSCynth (Ours)	**.68** ± .05	.31 ± .05
145	Real - Lesions Only	.67 ± .05**	**.33** ± .04
	Real - Balanced	.68 ± .04**	.32 ± .06
	LesionSCynth (Ours)	**.71** ± .02	.31 ± .02

References

De Leener, B., Lévy, S., Dupont, S. M., Fonov, V. S., Stikov, N., Louis Collins, D., Callot, V., & Cohen-Adad, J. (2017). SCT: Spinal Cord Toolbox, an open-source software for processing spinal cord MRI data. *NeuroImage*, *145*. https://doi.org/10.1016/j.neuroimage.2016.10.009

Gros, C., De Leener, B., Badji, A., Maranzano, J., Eden, D., Dupont, S. M., Talbott, J., Zhuoquiong, R., Liu, Y., Granberg, T., Ouellette, R., Tachibana, Y., Hori, M., Kamiya, K., Chougar, L., Stawiarz, L., Hillert, J., Bannier, E., Kerbrat, A., ... Cohen-Adad, J. (2019). Automatic segmentation of the spinal cord and intramedullary multiple sclerosis lesions with convolutional neural networks. *NeuroImage*, *184*. https://doi.org/10.1016/j.neuroimage.2018.09.081

Isensee, F., Wald, T., Ulrich, C., Baumgartner, M., Roy, S., Maier-Hein, K., & Jaeger, P. F. (2024, April 15). *nnU-Net Revisited: A Call for Rigorous Validation in 3D Medical Image Segmentation*. arXiv: 2404.09556 [cs]. https://doi.org/10.48550/arXiv.2404.09556

Philpott, C., & Brotchie, P. (2011). Comparison of MRI sequences for evaluation of multiple sclerosis of the cervical spinal cord at 3T. *European Journal of Radiology*, *80*(3). https://doi.org/10.1016/j.ejrad.2010.09.031

Walsh, R., Gaubert, M., Meurée, C., Hussein, B. R., Kerbrat, A., Casey, R., Combès, B., & Galassi, F. (2024). Multi-Sequence Learning for Multiple Sclerosis Lesion Segmentation in Spinal Cord MRI. In M. G. Linguraru, Q. Dou, A. Feragen, S. Giannarou, B. Glocker, K. Lekadir, & J. A. Schnabel (Eds.), *Medical Image Computing and Computer Assisted Intervention – MICCAI 2024*. Springer Nature Switzerland. https://doi.org/10.1007/978-3-031-72114-4_46

Walsh, R., Meurée, C., Kerbrat, A., Masson, A., Hussein, B. R., Gaubert, M., Galassi, F., & Combès, B. (2023). Expert Variability and Deep Learning Performance in Spinal Cord Lesion Segmentation for Multiple Sclerosis Patients. *2023 IEEE 36th International Symposium on Computer-Based Medical Systems (CBMS)*. https://doi.org/10.1109/CBMS58004.2023.00263

Walsh, R., & Tardy, M. (2023). A Comparison of Techniques for Class Imbalance in Deep Learning Classification of Breast Cancer. *Diagnostics*, *13*(1). https://doi.org/10.3390/diagnostics13010067
